# Diclofenac N-Derivatives as Therapeutic Agents with Anti-Inflammatory and Anti-Cancer Effect

**DOI:** 10.3390/ijms22105067

**Published:** 2021-05-11

**Authors:** Alberto Galisteo, Fatin Jannus, Amalia García-García, Houssam Aheget, Sara Rojas, José A. Lupiañez, Antonio Rodríguez-Diéguez, Fernando J. Reyes-Zurita, José F. Quílez del Moral

**Affiliations:** 1 Department of Organic Chemistry, Institute of Biotechnology, University of Granada, 18071 Granada, Spain; albertogapre@ugr.es; 2 Department of Biochemistry and Molecular Biology, University of Granada, C/Severo Ochoa s/n, 18071 Granada, Spain; fatin@correo.ugr.es (F.J.); jlcara@ugr.es (J.A.L.); 3 Department of Inorganic Chemistry, University of Granada, C/Severo Ochoa s/n, 18071 Granada, Spain; amaliagarcia@correo.ugr.es (A.G.-G.); srojas@ugr.es (S.R.); antonio5@ugr.es (A.R.-D.); 4 Centre for Genomics and Oncological Research, GENYO, C/Health Sciences Technology Park, Av. de la Illustration 114, 18016 Granada, Spain; houssam.aheget@genyo.es

**Keywords:** diclofenac, anticancer activity, anti-inflammatory activity, nitric oxide, drug development

## Abstract

A series of diclofenac N-derivatives (**2**, **4**, **6**, **8c**, **9c**, **10a**-**c**) were synthesized in order to test their anti-cancer and anti-inflammatory effects. The anticarcinogen activity has been assayed against three cancer cell lines: HT29, human colon cancer cells; Hep-G2, human hepatic cells; and B16-F10, murine melanoma cells. First, we determined the cytotoxicity of the different compounds, finding that the most effective compound was compound **8c** against all cell lines and both compounds **4** and **6** in human Hep-G2 and HT29 cell lines. Compounds **4** and **8c** were selected for the percentage of apoptosis determination, cell cycle distribution, and mitochondrial membrane potential measure because these products presented the lowest IC_50_ values in two of the three cancer cell lines assayed (B16-F10 and HepG2), and were two of the three products with lowest IC_50_ in HT29 cell line. Moreover, the percentages of apoptosis induction were determined for compounds **4** and **8c**, showing that the highest values were between 30 to 60%. Next, the effects of these two compounds were observed on the cellular cycle, resulting in an increase in the cell population in G2/M cell cycle phase after treatment with product **8c**, whereas compound **4** increased the cells in phase G0/G1, by possible differentiation process induction. Finally, to determine the possible apoptosis mechanism triggered by these compounds, mitochondrial potential was evaluated, indicating the possible activation of extrinsic apoptotic mechanism. On the other hand, we studied the anti-inflammatory effects of these diclofenac (DCF) derivatives on lipopolysaccharide (LPS) activated RAW 264.7 macrophages-monocytes murine cells by inhibition of nitric oxide (NO) production. As a first step, we determined the cytotoxicity of the synthesized compounds, as well as DCF, against these cells. Then, sub-cytotoxic concentrations were used to determine NO release at different incubation times. The greatest anti-inflammatory effect was observed for products **2, 4, 8c, 10a, 10b,** and **9c** at 20 µg·mL^−1^ concentration after 48 h of treatment, with inhibition of produced NO between 60 to 75%, and a concentration that reduces to the 50% the production of NO (IC_50 NO_) between 2.5 to 25 times lower than that of DCF. In this work, we synthesized and determined for the first time the anti-cancer and anti-inflammatory potential of eight diclofenac N-derivatives. In agreement with the recent evidences suggesting that inflammation may contribute to all states of tumorigenesis, the development of these new derivatives capable of inducing apoptosis and anti-inflammatory effects at very low concentrations represent new effective therapeutic strategies against these diseases.

## 1. Introduction

Non-steroidal anti-inflammatory drugs (NSAID) are compounds that produce anti-inflammatory effects, reducing pain, fever, and inflammation. The main mechanism of action of NSAID is the inhibition of cyclooxygenases (COX-1 and -2), enzymes related to the synthesis of key biological mediators (i.e., prostaglandins) involved in inflammation processes [1]. Particularly, diclofenac (DCF) is a well-known and common NSAID used to treat pain and inflammatory diseases, such as rheumatoid arthritis, post-operative traumatic pain, migraine, or fever. Like other NSAIDs, DCF is believed to work via inhibition of prostaglandin synthesis by blocking both COX-1 and -2. Other anti-inflammatory mechanisms proposed for the action of DCF are the inhibition of the thromboxane-prostanoid receptor, reduction of arachidonic acid release and uptake [2], protection on leukocyte-endothelium interactions, inhibition of lipoxygenase enzymes, and activation of nitric oxide-cGMP (3′,5′-cyclic guanosine monophosphate) antinociceptive pathway [1,3]. One example of the activity of DCF is the inhibition of over 93% of the phospholipase A2 enzymes (PLA2) activity, enzyme promoting inflammation via eicosanoids production, and direct activation of pro-inflammatory cells in patients with acute pancreatitis [4].

Recent evidence suggests that inflammatory actions can contribute to all tumorigenesis states [5]. Inflammation is also involved in critical steps of metastasis due to the production of angiogenic factors and cell migration [6]. Particularly, DCF presents an important range of actions, which are of interest in an oncological context, displaying a range of effects on inflammation, immune system response, angiogenic cascade, chemo-sensitivity in tumoral cells, and tumor metabolism. Different molecular mechanisms have been proposed for the anti-cancer action of DCF, some of which are common with other NSAIDs drugs: inhibition of COX-2 and decrease of PGE2 levels. High levels of this PGE2 were found in different types of cancer associated with chronic inflammation, providing a pro-tumor microenvironment [7]. Particularly in processes related to reducing PGE2 synthesis, DCF has shown to delay tumor growth and angiogenesis in BALB/c injected with C-26 adenocarcinoma cells [8] and reduce tumor growth volumes in the murine model [9].

Several in vitro and in vivo studies have demonstrated the antitumor effect of DCF. DCF inhibited cell growth via apoptosis in different cell lines, such as neuroblastoma cells (half maximal inhibitory concentration, IC_50_ = 30–178 µg·mL^−1^) [10], ovarian cancer cells SKOV-3, CAOV-3, SW626 and 36M2 (IC_50_ = 6–60 µg·mL^−1^) [11], and HEY and OVACAR-5 (IC_50_ = 15 µg·mL^−1^) [12], glioblastoma cells HTZ-349, U87MG and A172 (IC_50_ = 15–60 µg·mL^−1^) [13], or Hep-G2 cells (IC_50_ = 50 µg·mL^−1^) [14]. In vivo studies have demonstrated that DCF promoted a significant growth inhibitory effect on different tumors, such as C57/BL6 mice inoculated with B16 melanoma cells [15], rats carrying neuroblastoma xenografts SH-SY5Y [16], mice SK-N-AS neuroblastoma cells injected [9], mice with implanted fibrosarcoma [17], or BALB/c and CB6F1 mice [18]. When administered combined with other drugs, such as the anti-angiogenic drug TL-118, DCF induces the partial or complete remission of tumors in mice injected with C-26 adenocarcinoma cells [19], or SK-N-AS neuroblastoma cells [20].

Some in vitro reported studies evidenced the pro-apoptotic role of DCF in cancer. For example, it has been demonstrated that DCF induces DNA fragmentation and apoptosis, with caspases activation and cytochrome *c* release, associated with reactive oxygen species (ROS) increase and inhibition of Akt phosphorylation via phosphoinositide-3-kinase (PI3K) in HL-60 leukemia cells [21]. DCF has been shown to promote DNA fragmentation and caspases activation in neuroblastoma cells [10], increase ROS, activation of caspases-9 and -3, and reduce Bcl-2/Bax ratio and cytochrome *c* release in A2058 and SAN melanoma cells [22]. In leukemia cells HL-60, THP-1, and samples of acute myeloid leukemia patients, DCF induced apoptosis through activator protein-1 (AP-1) transcription factors (c-Jun, JunB, and Fra-2), induction of growth arrest and DNA damage-45α protein (GADD45α), and activation of c-Jun N-terminal kinase (JNK) [23].

Although DCF has been proved to be an effective analgesic or anti-pyretic in the treatment of cancer-related pain, this drug exhibits limitations and adverse effects towards gastrointestinal irritation, ulceration, platelet dysfunction, and hepatoxicity [24]. In this context, we have synthesized a series of DCF N-derivatives with the final aim of improving the biological properties of DCF as an anti-cancer and anti-inflammatory drug. Cytotoxicity studies using three cancer cell lines, namely B16-F10 murine melanoma cells, Hep-G2 hepatoma cells, and HT29 colon cancer cells, demonstrated that six of the as-synthesized compounds (**2**, **4**, **6**, **8c**, **9c,** and **10c**) showed an important improvement in their cytotoxicity activity with regard to DCF, reaching IC_50_ values between 13 to 48 µg·mL^−1^. Compounds **4** and **8c** were selected, and their apoptosis properties were assayed in Hep-G2 cell line with the percentage of apoptosis between 15 to 60%. In order to determine the plausible apoptotic mechanism of these compounds, the mitochondrial membrane potential was measured. Finally, we investigated the anti-inflammatory properties of synthetized compounds by means of the inflammation process induced in RAW 264.7 macrophages murine cells by bacterial lipopolysaccharide (LPS).

## 2. Results and Discussion

### 2.1. Synthesis of DCF Derivatives

**Scheme 1:**
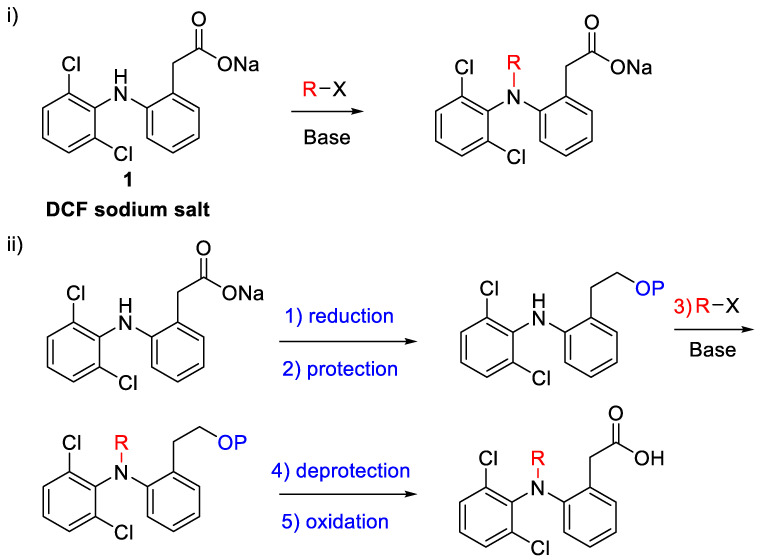
Two approaches to DCF N-derivatives.

Although the number of DCF analogs synthesized is overwhelming [25,26,27,28], the number of derivatives involving functionalization of the secondary amine of DCF is much more limited [29,30,31,32,33,34,35,36,37]. Scheme 1 shows the synthetic strategies employed for the synthesis of the DCF N-derivatives.

**Scheme 2:**
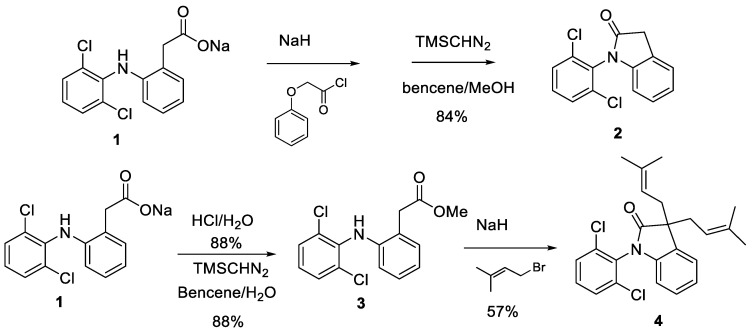
Synthesis of lactam **2** and its diisopentenyl derivative **4**.

We first tried the direct functionalization of DCF by reacting its sodium salt (**1**) with different electrophiles in the presence of NaH or K_2_CO_3_. Invariably the main reaction product was lactam **2** [38] (Scheme 2). When the corresponding methyl ester (**3**) was used as a starting material and isopentenyl bromide was used as electrophile, the dialkylated lactam **4** was obtained, again via lactam **2** (Scheme 2).

**Scheme 3:**
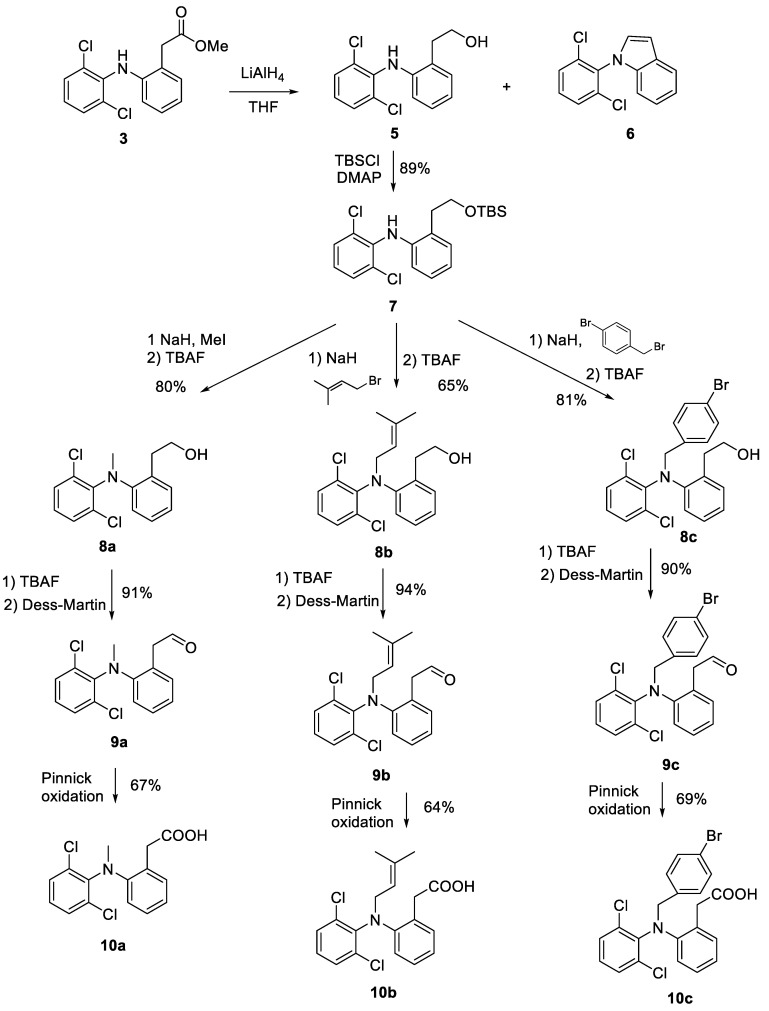
Synthesis of DCF N-derivatives.

In our second approach, we envisaged that the reduction of the phenylacetic acid of DCF to the corresponding primary alcohol not only would avoid the lactamization side reaction but also would enable the generation of N-functionalized derivatives with different oxidation degree. Following this approach, LAH reduction of methyl ester **3 [39]** to obtain alcohol **5** was achieved following the procedure described by Kelly et al. [40]. Together with **5**, variable minor amounts of the previously unreported indol derivative **6** were produced in this reduction (Scheme 3). Nucleophilic attack of the secondary amine to the aldehyde intermediate of the reduction process followed by a loss of water was proposed to rationalize the formation of **6**.

Continuing with our synthetic route, treatment of **5** with tert-butyldimethylsilyl chloride (TBSCl) in the presence of 4-dimethylaminopyridine (DMAP) led uneventfully to silyl derivative **7**. Gratifyingly the N-derivatization proceeds satisfactorily using NaH as a base and isopentenyl bromide, methyl iodide, and benzyl bromide to produce **8a**-**8c** after tetra-*n*-butylammonium fluoride (TBAF) deprotection of the silyl protecting group. Soft heating to 50 °C was necessary for the reaction to go to completion after approximately 6 h. Finally, the generation of the corresponding aldehyde derivatives was achieved using Dess-martin oxidations [41], whereas the generation of the targeted phenylacetic acids **10a [37]**, **10b** and **10c** involved the oxidation the corresponding aldehydes via Pinnick oxidation [42] (Scheme 3).

### 2.2. Anti-Carcinogenic Activity

#### 2.2.1. Cancer Cells Proliferation Studies

The eight DCF N-derivatives (**2**, **4**, **6**, **8c**, **9c**, **10a-c**) and their precursor (DCF) were assayed on B16-F10 murine melanoma cells, HT29 colon cancer cells, and Hep-G2 hepatoma cells, and IC_50_ values were determined (Table 1). In addition, we estimated the concentration required for 20 and 80% growth inhibition (IC_20_ and IC_80_, respectively). These concentrations were used for the rest of the assays.

**Figure 1:**
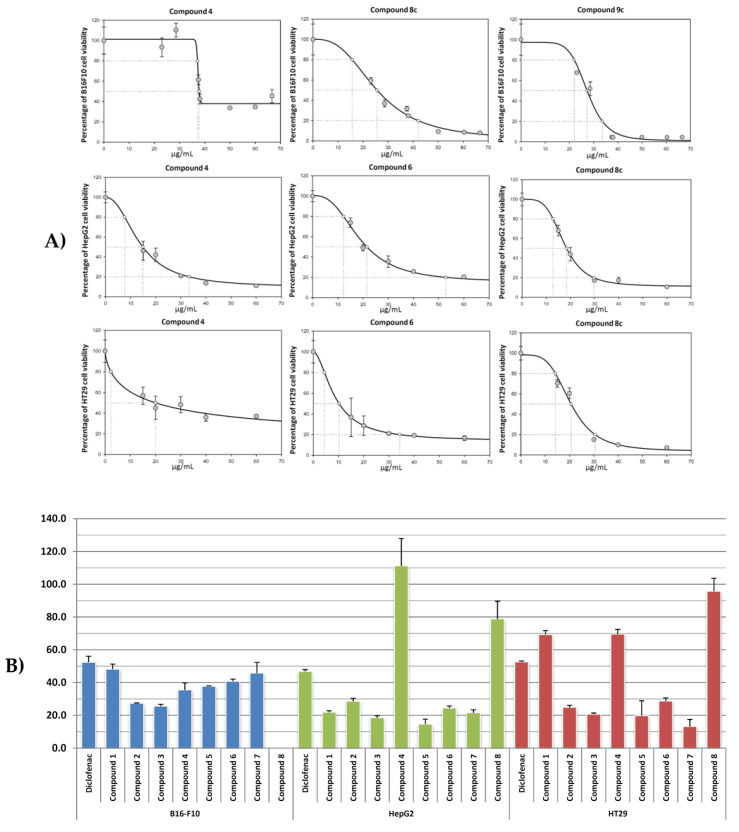
(**A**) Effects of compounds **4**, **8c,** and **9c**, on the viability of cell B16-F10, and compounds **4**, **6,** and **8c** on cells HT29 and Hep-G2, after treatment for 72 h in a range of 0 to 100 μg/mL. Each point represents the mean value ± S.D. of at least two independent experiments performed in triplicate. (**B**) IC_50_ values (μg·mL^−1^) of compounds **2**, **4**, **6**, **8c**, **9c**, **10a**-**c,** and DCF on B16-F10, Hep-G2 and HT29 cancer cells.

All tested products showed cytotoxic activity in the conditions assayed (Figure 1A), most of the compounds showed IC_50_ values between 25 to 48 μg·mL^−1^ in HT29 cells, 14 to 29 μg·mL^−1^ in Hep-G2 and HT29 cells. Except for compounds **10a**, **10b,** and **10c**, all synthetized compounds displayed lower IC_50_ values than DCF (IC_50_ = 52.5, 46.9, and 52.6 μg·mL^−1^ against B16-F10, Hep-G2, and HT29 cells, respectively). Particularly, derivatives **8c**, **9c**, **4** and **6** were between two to four times more cytotoxic than their precursor DCF, with IC_50_ values of 25.6 and 27.4 μg·mL^−1^ against B16-F10 cells for **8c** and **9c**, 18.5 and 14.6 μg·mL^−1^ against Hep-G2 cells for **8c** and **4**, and 19.8 and 13.2 μg·mL^−1^ against HT29 cells for **4** and **6**, respectively.

In general terms, the most effective compound was compound **8c** in all cell lines, and compounds **4** and **6** in Hep-G2 and HT29 cells lines, with IC_20_ values between 4 to 21 μg·mL^−1^, IC_50_ values between 13 to 27 μg·mL^−1^, and IC_80_ values between 30 to 42 μg·mL^−1^. These compounds were more effective than DCF in the three lines assayed, being between 1.4 to 2.1 times more cytotoxic than DCF in B16-F10 cells, between 2.2 to 3.2 times in Hep-G2 cells, and between 2.5 to 4.0 times in HT29 cells (Figure 1B). The lowest IC_50_ values were reached by compound **8c** in B16-F10 cells, compound **4** in Hep-G2 cells, and compound **6** in HT29 colon cancer cells.

Compounds possessing a 2-(2-((4-bromobenzyl)(2,6-dichlorophenyl)amino)phenyl moiety, namely compounds **8c**, **9c** and **10c**, showed good results in the cytotoxicity induced in the three cell lines, except for compound **10c** in HT29 cells. Compound **10b** resulted in being the least cytotoxic compound in the three cell lines assayed. Compounds with indolin substituent, **2**, **4**, and **6**, also presented good results in all cell lines assayed. Finally, compound **10a** did not induce cytotoxicity in the lines assayed. These effects could be related to a rise of the polarity of the complete molecule, without increasing the molecular weight or molecular volume, since probably most of these products performed their biological activity through the cellular membrane, which is prevented by excessively large molecular volumes.

A limited number of articles have been found describing the anti-cancer potential of DCF N derivatives. Thus, the cytotoxicity of the several oxadiazole DCF derivatives was analyzed in NIH 3T3 fibroblast cells. The oxadiazole derivative of diclofenac showed a value of IC_50_ = 32.0 ± 0.6 µg·mL^−1^, whereas the phenacyl derivative of 1,3,4-oxadiazole diclofenac showed an IC_50_ = 76.7 ± 6.0 µg·mL^−1^ [43].

Compounds **4** and **8c** were selected since they showed the lowest IC_50_ values in two out of the three cancer cell lines assayed (B16-F10 and HepG2), and were two of the three products with lowest IC_50_ in HT29 cell line. Compound **6** was not chosen because it showed a low IC_50_ value in HT29 cell line, and this value is very similar to that found for compound **4** in HepG2 cells. These products were selected for the percentage of apoptosis determination, cell cycle distribution, and mitochondrial membrane potential measure. All these assays were realized in HepG2 hepatoma cell line by flow cytometry analysis in a fluorescence-activated cell sorter. 

#### 2.2.2. Characterization of Apoptotic Effects

**Figure 2:**
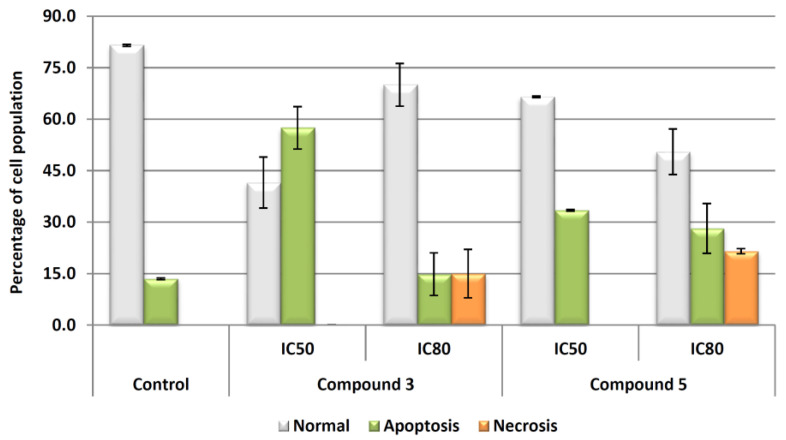
Flow cytometry results after exposure of Hep-G2 cells to DCF and the derivatives **4** and **8c** for 72 h at IC_50_ and IC_80_ concentrations. Apoptotic cells (green bars) were positive in annexin V, necrotic cells (orange bars) were positive in propidium iodide. Values are expressed as means ± S.E.M. of at least two experiments in duplicate.

The two compounds previously selected, **4** and **8c**, showed clearly apoptotic effects in treated cells, with total apoptosis between 30 and 57% at IC_50_ concentrations and between 15 to 35% at IC_80_ concentrations for products **8c** and **4**, respectively (Figure 2). The high percentage of necrosis (15%) for compound **8c** could be explained by a possible faster induction of apoptosis in response to product **8c**, and it could be related to the increase of the cell population in G2/M phase, as a consequence of apoptosis mechanism activation.

#### 2.2.3. Cell Cycle Arrest and Distribution

**Figure 3:**
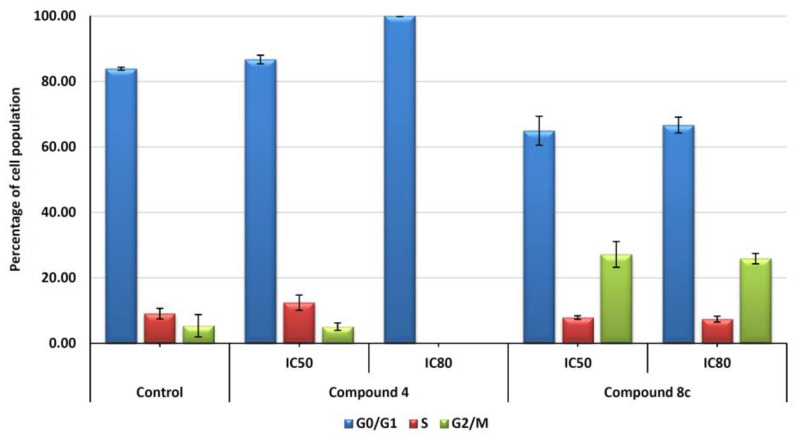
Cell-cycle phase representation (%) with respect to untreated control cells. Hep-G2 cells were treated with compounds **4** and **8c** for 72 h at IC_50_ and IC_80_ concentrations. Cells in the G0/G1 phase (blue bars), S phase (orange bars), and G2/M phase (green bars) were counted. Values represent means ± S.E.M. of at least two independent experiments performed in triplicate.

Further, we have investigated the effect of compounds **4** and **8c** on cell-cycle distribution in order to determine the possible cytostatic effects related to the cytotoxic response. Hep-G2 cells were treated with the selected products **4** and **8c** at both IC_50_ and IC_80_ concentrations. Flow cytometry was used to measure DNA ploidy as well as alterations in cell-cycle profiles (see the experimental section for further details). DNA histogram analysis (Figure 3) revealed that treatment with compound **8c** with both concentrations (IC_50_ and IC_80_) increase cell population at the G2/M phase compared with untreated control cells. This phenomenon might be a consequence of apoptosis induction, with an increase of 21% of cells in this cell cycle phase. On the other hand, treatment with product **4** produced an important increase in G0/G1 phase at IC_80_ concentration, reaching 100% of cells in this cell cycle phase. In this case, this high value could be due to a possible differentiation process activated in response to product **4**. Future assays will be necessary to confirm this point.

### 2.3. Effects on Changes in Mitochondrial Membrane Potential

**Figure 4:**
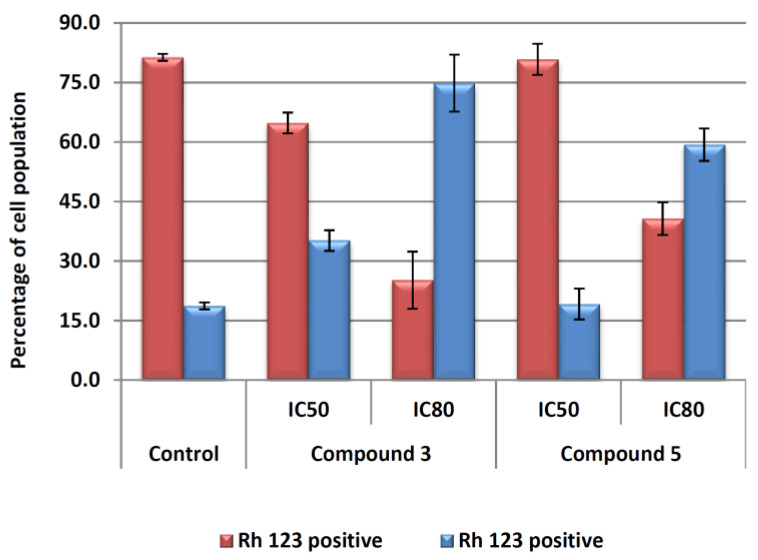
Flow-cytometry analysis of Rh123 staining after exposure of Hep-G2 cells to compounds **4** and **8c** for 72 h at IC_50_ and IC_80_ concentrations. Rh123 positive cells (red bars) and Rh123 negative cells (blue bars) were counted. Values are expressed as means ± S.E.M. of at least two experiments in duplicate.

The treatment with both compounds **4** and **8c** produced changes in the mitochondrial membrane potential (MMP, Figure 4). For compound **8c** at concentrations IC_50_ and IC_80_, 35 and 75% of cells lost MMP (Rh123 negative). Regarding compound **4**, there were practically no changes with respect to untreated control cells at IC_50_ concentration. However, at IC_80_ concentration, the percentage of cells with loss of MMP was about 60%.

These results are in agreement with those found in the apoptosis and cell cycle analysis. In both cases, the treatment produced apoptosis without loss of MMP at IC_50_ concentration, probably due to the activation of the extrinsic apoptosis mechanism. At IC_80_ concentration, both products produced the loss of MMP, probably because of the secondary activation of the intrinsic apoptotic pathway. The loss of MMP was faster in response to compound **8c** than to compound **4**, as showed the percentage of cells with loss of MMP at IC_50_ and IC_80_ concentrations, which agreed with the results of apoptosis and cell cycle obtained previously.

### 2.4. Effects DCF N-Derivatives in the Inflammatory Proces

Nitric oxide (NO), a molecule mediator in acute or chronic inflammation, is produced by constitutive or inducible nitric oxide synthases (iNOS). Its production is activated in response to pro-inflammatory signals as cytokines such as TNFα (tumor necrosis factor α) or INF-γ (interferon-γ), enterotoxin, or LPS (either bacterial lipopolysaccharide), in macrophages [44]. Here, the decrease of NO concentration has been used as an indirect marker of the inflammatory process [44,45]. In the inflammatory response process, NO is released as an intermediate or second messenger. RAW 264.7 murine macrophage cells are an especially indicated model in anti-inflammatory compounds screening studies since they produce the highest release of NO during the inflammatory response. In this study, the anti-inflammatory potential of DCF N-derivatives was analyzed by measuring nitrites in a cell culture medium since the nitrites concentration is proportional to the NO release. With this purpose, we measured the inhibition in NO production in activated LPS RAW 264.7 macrophage cells.

#### 2.4.1. Raw 264.7 Cell Viability

First, RAW 264.7 cell viability was assayed with compounds **2** [46], **4**, **6**, **8c**, **9c**, **10a-c,** and DCF to establish sub-cytotoxic concentrations and, thus, to assure that anti-inflammatory effects are a consequence of the anti-inflammatory activity of the compounds rather than their cytotoxicity. The concentration of products required for 50% growth inhibition was determined as previously described for B16-F10, Hep-G2, and HT29 cells. (see the experimental section for further details). The results showed similar cytotoxicity for all compounds at the conditions assayed (Table 2). Based on these results, sub-cytotoxic concentrations used in the determination of anti-inflammatory response were set at 5, 10, and 20 µg·mL^−1^.

#### 2.4.2. Nitric Oxide Production

**Figure 5:**
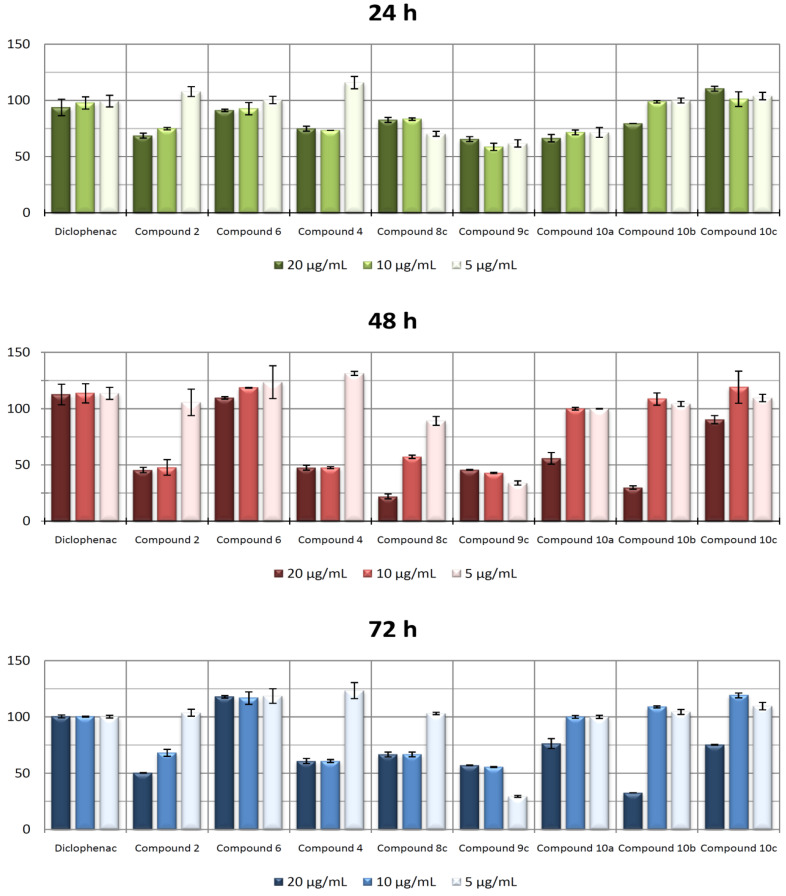
Effect of compounds **2**, **4**, **6**, **8c**, **9c**, **10a**-**c,** and DCF on the release of nitrites in RAW 264.7 macrophage murine cells. After activation of the inflammatory process, compounds were incubated for 24 h, 48 h, and 72 h at 5, 10, and 20 µg·mL^−1^ concentrations. The data represent the mean ± S.D. of at least two independent experiments performed in triplicate.

Firstly, macrophages RAW 264.7 were activated with LPS for 24 h. After this stimulation period, cells were incubated with compounds **2**, **4**, **6**, **8c**, **9c**, **10a**-**c,** and DCF for 72 h. Aliquots at different incubation times were taken, and nitrite concentration was determined (see the experimental section for further details). Our results showed that at the concentrations assayed (5, 10, and 20 µg·mL^−1^), all compounds produced inhibition of NO release higher than DCF with respect to the positive control (only LPS treated control cells, 100% release), except for compounds **6** and **10c** (Figure 5). After 24 h, NO release inhibition between 25 to 30% was achieved when using between 10 and 20 µg·mL^−1^ of products **2**, **4**, **8c**, **9c**, **10a,** and **10b**, and 5 µg·mL^−1^ of products **8c**, **9c,** and **10a**. The highest anti-inflammatory effect was reached with 20 µg·mL^−1^ of compounds **8c** and **10b** at 48 h, with 75% of NO inhibition. Compound **9c** showed the highest NO inhibition (60%) at the lower concentration (5 µg·mL^−1^). Products **2**, **4,** and **10a** showed an inhibition close to 50% at 10 and 20 µg·mL^−1^ concentrations. The results obtained at 72 h of incubation were similar to those found at 48 h. 

**Figure 6:**
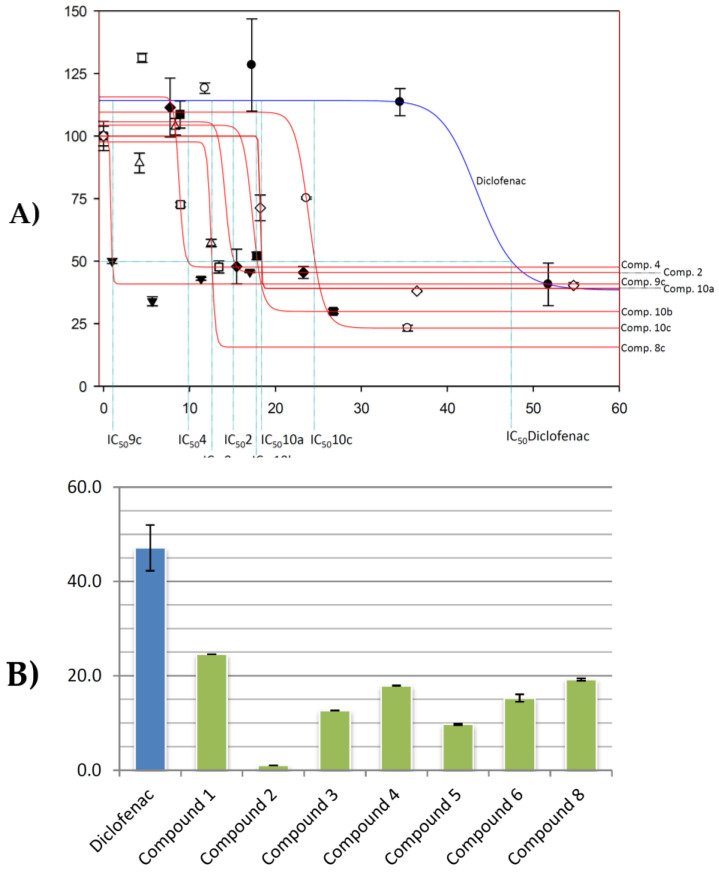
(**A**). Sigmoidal curves of the effect of compounds **2**, **4**, **6**, **8c**, **9c**, **10a**-**c** (red) and DCF (blue) on the release of nitrites in RAW 264.7 monocyte/macrophage murine cells activated with LPS. The values corresponding to IC_50 NO_ for each compound can be seen in the curves. The data represent the mean ± S.D. of at least two independent experiments performed in triplicate. (**B**). NO release-inhibitory effects. IC_50 NO_ μg·mL^−1^ concentrations for compounds **2**, **4**, **6**, **8c**, **9c**, **10a**-**c**, and DCF in RAW 264.7 monocyte/macrophage murine cells activated with LPS.

For a complete anti-inflammatory characterization of compounds, we calculated the concentration that reduces to 50% the production of NO (IC_50 NO_) at 48 h of cell incubation (Figure 6A). Our data showed that the IC_50 NO_ concentration of compound **9c** (IC_50 NO_ = 1.89 ± 0.11 µg·mL^−1^) was lower than those found for the rest of the compounds. This value was 25 times less than that found for DCF (IC_50 NO_ = 47.12 ± 4.85 µg·mL^−1^). Products **2**, **4**, **8c**, **10a,** and **10b** showed IC_50 NO_ values between 10 to 20 µg·mL^−1^. These data were between 2.5 to 4.5 times more effective than DCF. The IC_50 NO_ for compound **10c** was 24.57 ± 0.3 µg·mL^−1^, the highest found. In summary, all products assayed, except **6**, inhibited the inflammation process produced by LPS in RAW 264.7 murine macrophage-monocyte cells. The greatest anti-inflammatory effects were observed at 48 h of incubation. At 20 µg·mL^−1^, the products that showed greater inhibition of NO release were compounds **8c**, **9c,** and **10b**. However, according to IC_50 NO_ values, the products with greater effectiveness were products **4**, **8c,** and **9c** (Figure 6B).

Other DCF derivatives with potent inhibition of NO production have been synthesized, such as a number of oxadiazole derivatives, which presented IC_50 NO_ values ranging from 7.13 ± 1.0 µg·mL^−1^ to 14.8 ± 2.0 µg·mL^−1^. These results were obtained in LPS stimulated mouse macrophage J774.2 cell line [43]. Additionally, different DCF-thiadiazole, DCF -triazole, and DCF oxadiazole hybrids proved to show moderate to strong anti-inflammatory activity in acute carrageenan-induced paw edema in male adult albino rats, in comparison with DFC [47]. Furthermore, a series S-substituted phenacyl 1,3,4-oxadiazoles and Schiff bases derived from DCF were tested in vivo for their anti-inflammatory activity [48]. Eight of these derivatives showed significant anti-inflammatory activity in the carrageenan-induced rat paw edema model. 

### 2.5. Conclusions

The design of new drugs based on proven bioactive products, as DCF, is a new and promising strategy to find new compounds with high and enhanced biochemical properties. Particularly, the functionalization of the secondary amine in the development of new derivatives has demonstrated to be an efficient strategy to achieve several interesting bioactive compounds. N-derivatives **2**, **4**, **6**, **8c, 9c,** and **10c** showed an improved cytotoxic effect against B16-F10, Hep-G2, and HT29 cancer cells than pristine DCF. Particularly, compounds **4** and **8c** were found to be the most effective against all cell lines, with IC_50_ values between 13 to 27 μg·mL^−1^. The selected compounds, **4** and **8c**, showed clearly apoptotic effects in treated cells, with a total apoptosis between 30 and 57% at IC_50_ concentrations. DNA histogram analysis revealed that treatment with IC_50_ concentration of compound **8c** produced an increase in the cell population at the G2/M phase compared with untreated control cells. On the contrary, when using IC_80_ concentration of product **4**, an important increase of in G0/G1 phase. Further, **8c** was able to produce changes in MMP (35% at IC_50_). Finally, the results obtained in the anti-inflammatory test showed that the anti-inflammatory response of **2**, **4**, **8c**, **10a**, and **10b** is from 2.5 to 4.5-fold better than DCF. Particularly, among all the tested compounds, after 48 h **4**, **8c** and **9c** (20 µg·mL^−1^) showed the greater inhibition of NO release. In conclusion, **8c** shows the most encouraging results and could be further exploited for developing new, improved anti-inflammatory and anti-cancer agents.

## 3. Materials and Methods

### 3.1. Chemistry

Silica gel 60 (35-70 μm) was used for flash column chromatography. NMR spectra were obtained in a Varian Direct-Drive 600 (^1^H 600 MHz/^13^C 150 MHz), Varian Direct-Drive 500 (^1^H 500 MHz/^13^C 125 MHz), and Varian Direct-Drive 400 (^1^H 400 MHz/^13^C 100 MHz). Accurate mass determinations were performed on a SYNAPT G2-Si Q-TOF mass spectrometer (Waters, Milford, MA, USA) equipped with high-efficiency T-Wave ion mobility and an orthogonal Z–spray™ electrospray ionization (ESI) source. MassLynx v.4.1 software was used for HRMS instrument control, peak detection, and integration. Reactions were monitored by TLC on 0.25 mm E. Merck silica gel plates (60F-254) visualized under UV light and by applying a phosphomolybdic acid solution in EtOH followed by heat. High-quality reagents were purchased at the highest quality that was commercially available and was used without further purification.

### 3.2. Synthesis of DCF N-Derivatives

#### 3.2.1. Preparation of DCF from Its Sodium Salt (**1**)

To a heated (50 °C) solution of DCF sodium salt (2 g, 6.76 mmol) in 500 mL of water, 2N HCl solution was added under stirring till pH = 6. The resulting precipitated was filtered to afford an 88% yield of DCF (1.75 g, 5.95 mmol).

#### 3.2.2. Treatment of **1** with Phenoxyacetyl Chloride in the Presence of NaH

To a solution of **1** (200 mg, 0.63 mmol) in 17 mL THF, under an argon atmosphere, was added 50.3 mg of NaH (1.26 mmol). Then, 0.17 mL of phenoxyacetyl chloride (1.26 mmol) were added dropwise. The resulting solution was stirred for 18 h, and then 25 mL of EtOAc were added, followed by 5 mL of saturated NH_4_Cl. The aqueous layer was diluted with water (15 mL) and extracted with MTBE (3 × 15 mL). The combined organic layers were washed with brine (3 × 25 mL) dried over anhydrous Na_2_SO_4_, and concentrated under reduced pressure. The crude product was purified by flash chromatography (H:MTBE, 1:1) to give a 84% yield of compound **2** (151 mg, 0.54 mmol) [38].

**Compound 2.** ^1^H NMR (500 MHz, CDCl_3_) δ 7.53 (d, *J* = 8.1 Hz, 2H), 7.39 (dd, *J* = 8.7, 7.6 Hz, 1H), 7.36 (bd, *J* = 7.4 Hz, 1H), 7.23 (td, *J* = 7.7, 1.2 Hz, 1H), 7.12 (td, *J* = 7.5, 1.1 Hz, 1H), 6.43 (dt, *J* = 8.0, 1.0 Hz, 1H), 3.80 (s, 2H) (Figure S1a). ^13^C NMR (125.1 MHz, CDCl_3_) δ 173.65 (C), 143.34 (C), 135.52 (2C), 130.87 (CH), 130.48 (C), 129.08 (2CH), 127.97 (CH), 124.87 (CH), 124.32 (C), 123.11 (CH), 109.16 (CH), 35.77 (CH_2_) (Figure S1b).

#### 3.2.3. Treatment of DCF (**5**) with Diazomethane

To a stirred solution of DCF **5** (296 mg, 1.00 mmol) in 4.1 mL of benzene and 1 mL of MeOH, 0.6 mL (1.2 mmol) of a 2 M solution of TMSCH_2_N_2_ in hexanes were added dropwise. After consumption of the starting product (30 min), the mixture was concentrated under reduced pressure. Purification by flash chromatography (H:MTBE, 4:1) afforded 278 mg (88% yield) of ester **3** (278 mg, 0.9 mmol). The spectroscopic data of compound **3** coincide with those reported in the literature [39].

#### 3.2.4. Treatment of Compound **3** with Isopentenyl Bromide in the Presence of NaH

To a stirred solution of ester **3** (165 mg, 0.53 mmol) in 5 mL THF, under an argon atmosphere, was added 21.2 mg of NaH (0.53 mmol). Then, 0.1 mL of isopentenyl bromide were added dropwise. The resulting solution was stirred for 30 min, and then 15 mL of MTBE were added, followed by 3 mL of saturated NH_4_Cl. The aqueous layer was diluted with water (15 mL) and extracted with MTBE (3 × 15 mL). The combined organic layers were washed with brine (3 × 20 mL) dried over anhydrous Na_2_SO_4_, and concentrated under reduced pressure. The crude product was purified by flash chromatography (H:MTBE, 6:1) to afford 125 mg of compound **4** (0.30 mmol, 57%).

**Compound 4.** ^1^H NMR (500 MHz, CDCl_3_) δ 7.50 (d, *J* = 8.1 Hz, 2H), 7.36 (dd, *J* = 8.6, 7.6 Hz, 1H), 7.30 (dd, *J* = 7.4, 0.8 Hz, 1H), 7.19 (td, *J* = 7.7, 1.3 Hz, 1H), 7.10 (td, *J* = 7.5, 1.1 Hz, 1H), 6.37 (dt, *J* = 7.7, 0.8 Hz, 1H), 5.03 (thept, *J* = 7.6, 1.5 Hz, 2H), 2.72 (dd, *J* = 14.2, 8.0 Hz, 2H), 2.64 (dd, *J* = 14.1, 7.0 Hz, 2H), 1,57 (bs, 6H), 1,57 (bs, 6H) (Figure S1d). ^13^C NMR (125.1 MHz, CDCl_3_) δ 178.35 (C), 142.01 (C), 135.59 (2C), 134.85 (2C), 131.93 (C), 130.73 (C), 130.41 (CH), 128.99 (2CH), 127.49 (CH), 123.88 (CH), 122.53 (CH), 118.53 (2CH), 108.58 (CH), 53.64 (C), 36.02 (2CH_2_), 25.94 (2CH_3_), 18.06 (2CH_3_) (Figure S1e). HRMS TOF (ESI+) m/z calculated for C_24_H_26_ClNO [M + H]^+^ 414.1391, found 414.1399.

#### 3.2.5. Reduction of DCF Methyl Ester **3** with Lithium Aluminum Hydride (LAH)

To a solution of DCF methyl ester (**3**) (1 g, 3.4 mmol) in 25 mL THF, cooled at 0 °C and under an argon atmosphere, was added 384 mg of LAH (10 mmol). The resulting solution was stirred for 1 h and then diluted with methyl *tert*-butyl ether (MTBE) (100 mL) and washed with saturated NH_4_Cl (15 mL). The aqueous layer was extracted with MTBE (3 × 30 mL). The combined organic layers were washed with brine (3 × 25 mL), dried over anhydrous Na_2_SO_4_, and concentrated under reduced pressure. Purification by flash chromatography (H:MTBE, 1:1) afforded 190 mg (0.68 mmol, 20%) of compound **6** and 550 mg (1.97 mmol, 58%) of compound **5** [40].

**Compound 5**: ^1^H NMR (600 MHz, CDCl_3_) δ = 7.36 (d, *J* = 8.1 Hz, 2H), 7.21 (dd, *J* = 7.4, 1.5 Hz, 1H), 7.10 (td, *J* = 7.7, 1.7 Hz, 1H), 7.00 (t, *J* = 8.1 Hz, 1H), 6.96 (td, *J* = 7.4, 1.1 Hz, 1H), 6.51 (dd, *J* = 8.0, 1.1 Hz, 1H), 4.04 (t, *J* = 5.9 Hz, 2H), 3.04 (t, *J* = 5.9 Hz, 2H) (Figure S1g). ^13^C NMR (151 MHz, CDCl_3_) δ = 142.64 (C), 137.74 (C), 130.63 (CH), 129.84 (CH), 128.88 (CH), 128.71 (C), 126.99 (CH), 124.03 (CH), 121.62 (C), 116.88 (CH), 64.17 (CH_2_), 34.85 (CH_2_) (Figure S1h).

**Compound 6**: ^1^H NMR (500 MHz, CDCl_3_) δ 7.80 (m, 1H), 7.56 (d, *J* = 8.1 Hz, 2H), 7.40 (t, *J* = 8.1 Hz, 1H), 7.28 (m, 2H), 7.19 (d, *J* = 3.3 Hz, 1H), 7.06–7.01 (m, 1H), 6.84 (d, *J* = 3.4 Hz, 1H) (Figure S1j). ^13^C NMR (125.1 MHz, CDCl_3_) δ 136.21 (C), 135.64 (2C), 134.86 (C), 130.09 (CH), 128.95 (2CH), 128.33 (C), 128.06 (CH), 122.60 (CH), 121.15 (CH), 120.58 (CH), 110.29 (CH), 103.87 (CH). HRMS TOF (ESI+) m/z calculated for C_14_H_10_Cl_2_N [M + H]^+^ 262.0190, found 262.0177 (Figure S1k).

#### 3.2.6. Treatment of Alcohol **6** with TBSCl

To a stirred solution of compound **6** (337 mg, 1.2 mmol) in 20 mL of dry dichloromethane (DCM) were added under argon atmosphere 500 mg (4.1 mmol) of 4-(dimethylamino)pyridine (DMAP) and tert-butyldimethylsilyl chloride (TBSCl) (328 mg, 2.2 mmol). After consumption of the starting product (45 min), the mixture was diluted with 150 mL of DCM and washed with 1 N HCl (3 × 20 mL), Na_2_CO_3_ (3 × 30 mL) and brine (3 × 30 mL). The organic layer was dried over anhydrous Na_2_SO_4_ and concentrated under reduced pressure. The resulting reaction crude was flash chromatographed (H:MTBE, 5:1) to afford 423 mg (1.1 mmol) of compound **7** (89%).

**Compound 7**: ^1^H NMR (500 MHz, CDCl_3_) δ 7.36 (d, J = 8.1 Hz, 2H), 7.20 (dd, *J* = 7.4, 1.5 Hz, 1H), 7.07 (td, *J* = 7.7, 1.6 Hz, 1H), 7.01 (t, *J* = 8.1 Hz, 1H), 6.94 (td, *J* = 7.4, 1.2 Hz, 1H), 6.49 (dd, *J* = 8.0, 1.0 Hz, 1H), 3.99 (t, *J* = 6.7 Hz, 2H), 3.01 (t, *J* = 6.7 Hz, 2H), 0.88 (s, 9H), 0.06 (s, 6H) (Figure S1l). ^13^C NMR (125.1 MHz, CDCl_3_) δ 142.21 (C), 137.80 (C), 130.56 (CH), 130.09 (2C), 128.86 (2C), 128.59 (C), 126.78 (CH), 124.17 (CH), 121.60 (CH), 117.04 (CH), 63.91 (CH_2_), 35.32 (CH_2_), 26.04 (3CH_3_), 18.60 (C), -5.22 (2CH_3_). HRMS TOF (ESI+) m/z calculated for C_20_H_28_Cl_2_NOSi [M + H]^+^ 397.1317, found 397.1308 (Figure S1m).

#### 3.2.7. Treatment of Compound **7** with Methyl Iodide in the Presence of NaH: Synthesis of **8a**

To a stirred solution of compound **7** (395 mg, 1 mmol) in 18 mL THF, under an argon atmosphere, was added 75 mg of NaH (3.1 mmol). After stirring for 30 min, 0.15 mL (2.4 mmol) of methyl iodide was added dropwise, and the resulting solution was stirred for 1 h. The mixture was then cooled at 0 °C and diluted with 150 mL of MTBE. Then 5 mL of saturated NH_4_Cl were added dropwise. The aqueous layer was diluted with water (15 mL) and extracted with MTBE (3 × 20 mL). The combined organic layers were washed with brine (3 × 40 mL) dried over anhydrous Na_2_SO_4_, and concentrated under reduced pressure. The resulting crude product was dissolved in 8 mL of dry THF, and 700 mg of TBAF was added to the resulting solution at room temperature and under argon atmosphere. After stirring for 30 min, the reaction mixture was diluted with 150 mL of EtOAc, washed with brine (3 × 35 mL), dried over anhydrous Na_2_SO_4_ and concentrated in vacuo. The reaction crude was purified by flash chromatography (H:MTBE, 1:1) to afford 238 mg of compound **8a** (0.8 mmol, 80%). 

**Compound 8a**. ^1^H NMR (600 MHz, CDCl_3_) δ 7.34 (d, *J* = 8.0 Hz, 2H), 7.25 (td, *J* = 7.4, 1.4 Hz, 1H), 7.21 (dd, *J* = 8.2, 1.4 Hz, 1H), 7.13 (dd, *J* = 7.5, 1.7 Hz, 1H), 7.10 (t, *J* = 8.0 Hz 1H), 6.98 (td, *J* = 7.4, 1.4 Hz, 1H), 3.55 (t, *J* = 6.9 Hz, 2H), 3.32 (s, 3H), 2.45 (t, *J* = 6.9 Hz, 2H) (Figure S1o). ^13^C NMR (151 MHz, CDCl_3_) δ 147.95 (C), 147.95 (C), 135.14 (2C), 130.78 (C), 129.78 (2CH), 129.05 (CH), 127.05 (CH), 126.93 (CH), 121.83 (CH), 120.39 (CH), 62.33 (CH_2_), 40.49 (CH), 35.06 (CH_2_). HRMS TOF (ESI+) m/z calculated for C_15_H_16_NOCl_2_ [M + H]^+^ 296.0699, found 296.0691 (Figure S1p).

#### 3.2.8. Dess-Martin Oxidation of **8a**

To a stirred solution of **8a** (100 mg, 0.34 mmol) in 15 mL of DCM under argon was added the Dess−Martin reagent (305 mg, 0.72 mmol). After stirring for 40 min at room temperature, the reaction was quenched with 10 mL of a saturated solution of Na_2_S_2_O_3_−NaHCO_3_ that was added slowly to the mixture. The organic layer was then washed with brine (3 × 20 mL), dried over anhydrous Na_2_SO_4_ and concentrated under reduced pressure. The crude product was purified by flash chromatography (H:MTBE, 2:1) to obtain 91 mg of **9a** (0.31 mmol, 91%).

**Compound 9a**. ^1^H NMR (600 MHz, CDCl_3_) δ 9.17 (t, *J* = 1.9 Hz, 1H), 7.36–7.33 (m, 1H), 7.33 (d, *J* = 8.1 Hz, 2H), 7.26 (d, *J* = 8.1 Hz, 1H), 7.10 (t, *J* = 8.1 Hz, 1H), 7.05–7.00 (m, 1.0 Hz, 2H), 3.31 (d, *J* = 1.9 Hz, 2H), 3.30 (s, 3H) (Figure S1y). ^13^C NMR (151 MHz, CDCl_3_) δ 199.56 (CHO), 148.57 (C), 143.59 (C), 135.35 (2C), 132.19 (CH), 129.93 (2CH), 128.20 (CH), 127.48 (CH), 123.41 (C), 121.98 (CH), 120.26 (CH), 46.65 (CH_2_), 40.27 (CH) (Figure S1z). HRMS TOF (ESI+) m/z calculated for C_15_H_14_NOCl_2_ [M + H]^+^ 294.0452, found 294.0442.

#### 3.2.9. Pinnick Oxidation of **9a**

Aldehyde **9a** (98 mg, 0.33 mmol) was dissolved in 7 mL of *tert*-butyl alcohol and 1.2 mL of 2-methyl-2-butene. A solution of sodium chlorite (85 mg, 0.94 mmol) and sodium dihydrogenphosphate dihydrate (95 mg, 0.61 mmol) in 1.5 mL of water was added dropwise over a 10 min period. The yellow reaction mixture was stirred at room temperature for 40 min. Volatile components were then removed under vacuum. The residue was dissolved in 30 mL of water and extracted with two 25 mL portions of MTBE. The combined organic layers were washed with brine, dried, and concentrated under reduced pressure. Purification by flash chromatography (H:MTBE, 10:1) afforded 69 mg of compound **10a [37]** (0.22 mmol, 67%). 

**Compound 10a**: ^1^H NMR (600 MHz, CDCl_3_) δ 7.31 (ta, *J* = 8,1 Hz, 1H), 7.29 (d, *J* = 8.1 Hz, 2H), 7.20 (d, *J* = 8.1 Hz, 1H), 7.13 (td, *J* = 6.8 Hz, 1H), 7.03 (t, *J* = 8.1 Hz, 1H), 7.01 (t, *J* = 7.4 Hz, 1H), 3.42 (s, 2H), 3.30 (s, 3H) (Figure S1h2). ^13^C NMR (151 MHz, CDCl_3_) δ 175.94 (COOH), 148.14 (C), 143.39 (C), 135.62 (2C), 132.09 (CH), 129.76 (2CH), 128.05 (CH), 127.28 (CH), 124.47 (C), 121.90 (CH), 120.47 (CH), 40.47 (CH), 36.85 (CH_2_) (Figure S1i2). HRMS TOF (ES+) m/z calculated for C_15_H_14_NO_2_Cl_2_ [M + H]^+^ 310.0402, found 310.0390. 

#### 3.2.10. Treatment of Compound 7 with Isopentenyl Bromide in the Presence of NaH: Synthesis of **8b**

To a stirred solution of compound **7** (675 mg, 1.65 mmol) in 25 mL THF, under argon atmosphere, was added 123 mg of NaH (5.1 mmol). After stirring for 30 min, 0.7 mL (0.95 mmol) of isopentenyl bromide were added dropwise, and the resulting solution was stirred for 6 h at 50 °C. The mixture was then cooled at 0 °C and diluted with 150 mL of MTBE. Then 15 mL of saturated NH_4_Cl were added dropwise. The aqueous layer was extracted with MTBE (3 × 30 mL). The combined organic layers were washed with brine (3 × 40 mL) dried over anhydrous Na_2_SO_4_ and concentrated under reduced pressure. The resulting crude product was dissolved in 25 mL of dry THF, and 1.8 mL of 1M TBAF solution in THF was added to the resulting solution at room temperature and under argon atmosphere. After stirring for 3 h the reaction mixture was diluted with 200 mL of EtOAc, washed with brine (3 × 50 mL), dried over anhydrous Na_2_SO_4_ and concentrated in vacuo. The reaction crude was purified by flash chromatography (H:MTBE, 9:1) to afford 384 mg of compound **8b** (1.1 mmol, 65%). 

**Compound 8b**. ^1^H NMR (500 MHz, CDCl_3_) δ 7.31 (d, *J* = 8.0 Hz, 2H), 7.23–7.17 (m, 2H), 7.10 (dd, *J* = 7.5, 1.5 Hz, 1H), 7.05 (dd, *J* = 8.3, 7.7 Hz, 1H), 6.95 (ddd, *J* = 7.6, 6.1, 2.3 Hz, 1H), 5.39 (thept, *J* = 6.13, 1.4 Hz, 1H), 4.32 (d, *J* = 6.2 Hz, 2H), 3.52 (t, *J* = 6.9 Hz, 2H), 2.41 (t, *J* = 6.9 Hz, 2H), 1.67 (bd, *J* = 1.4 Hz, 3H), 1.63 (bd, *J* = 1.4 Hz, 3H) (Figure S1q). ^13^C NMR (126 MHz, CDCl_3_) δ 146.70 (C), 143.49 (C), 135.31 (2C), 134.25 (C), 130.70 (CH), 129.89 (C), 129.77 (2CH), 126.72 (CH), 126.55 (CH), 121.97 (CH), 121.94 (CH), 121.55 (CH), 62.39 (CH_2_), 51.06 (CH_2_), 35.03 (CH_2_), 25.75 (CH_3_), 17.90 (CH_3_) (Figure S1r). HRMS TOF (ES+) m/z calculated for C_19_H_22_NOCl_2_ [M + H]^+^ 350.1078, found 350.1062.

#### 3.2.11. Dess-Martin Oxidation of **8b**

To a stirred solution of **8b** (60 mg, 0.17 mmol) in 5 mL of DCM under argon was added the Dess−Martin reagent (144 mg, 0.34 mmol). After stirring for 20 min at room temperature, the reaction was diluted with DCM (20 mL) and quenched with 2 mL of a saturated solution of Na_2_S_2_O_3_−NaHCO_3_ that was added slowly to the mixture. The organic layer was then washed with brine (3 × 10 mL), dried over anhydrous Na_2_SO_4_, and concentrated under reduced pressure. The crude product was purified by flash chromatography (H:MTBE, 2:1) to obtain 58 mg of **9b** (0.16 mmol, 94%). 

**Compound 9b**. ^1^H NMR (500 MHz, CDCl_3_) δ 9.18 (t, *J* = 2.0 Hz, 1H), 7.34–7.28 (m, 3H), 7.24 (d, *J* = 7.7 Hz, 1H), 7.08 (t, *J* = 8.1 Hz, 1H), 7.05–6.98 (m, 2H), 5.41 (bt, *J* = 6.3 Hz, 1H), 4.32 (d, *J* = 6.4 Hz, 2H), 3.30 (d, *J* = 2.1 Hz, 2H), 1.70 (bs, 3H), 1.64 (bs, 3H) (Figure S1b2). ^13^C NMR (126 MHz, CDCl_3_) δ 199.67 (C), 147.44 (C), 142.94 (C), 135.58 (2C), 134.66 (C), 132.12 (CH), 129.94 (2CH), 127.90 (CH), 127.16 (CH), 124.26 (C), 122.10 (CH), 121.88 (CH), 121.21 (CH), 51.02 (CH_2_), 46.64 (CH_2_), 25.76 (CH_3_), 17.91 (CH_3_) (Figure S1c2). HRMS TOF (ES+) m/z calculated for C_19_H_20_NOCl_2_ [M + H]^+^ 348.0922, found 348.0901.

#### 3.2.12. Pinnick Oxidation of **9b**

Aldehyde **9b** (243 mg, 0.71 mmol) was dissolved in 20 mL of *tert*-butyl alcohol and 3 mL of 2-methyl-2-butene. A solution of sodium chlorite (260 mg, 2.87 mmol) and sodium dihydrogenphosphate dihydrate (295 mg, 1.89 mmol) in 5 mL of water was added dropwise over a 20 min period. The yellow reaction mixture was stirred at room temperature for 50 min. Volatile components were then removed under vacuum. The residue was dissolved in 30 mL of water and extracted with two 50 mL portions of MTBE. The combined organic layers were washed with brine, dried, and concentrated under reduced pressure. Purification by flash chromatography (H:MTBE, 10:1) afforded 164 mg of compound **10b** (0.45 mol, 64%).

**Compound 10b**: ^1^H NMR (400 MHz, CDCl_3_) δ 7.24 (d, *J* = 8.0 Hz, 1H), 7.27–7.24 (m, 1H), 7.19 (dd, *J* = 8.3, 1.3 Hz, 1H), 7.12 (dd, *J* = 7.6, 1.7 Hz, 1H), 7.02–6.96 (m, 2H), 5.40 (thep, *J* = 6.4, 1.4 Hz, 1H), 4.32 (dt, *J* = 6.4, 1.3 Hz, 2H), 3.40 (s, 2H), 1.68 (d, *J* = 1.4 Hz, 3H), 1.62 (d, *J* = 1.4 Hz, 3H) (Figure S1k2). ^13^C NMR (101 MHz CDCl_3_) δ 176.83 (C), 147.11 (C), 142.60 (C), 135.90 (2C), 134.47 (C), 131.97 (CH), 129.73 (2CH), 127.70 (CH), 126.95 (CH), 125.30 (C), 121.99 (CH), 121.98 (CH), 121.38 (CH), 51.15 (CH_2_), 36.93 (CH_2_), 25.74 (CH_3_), 17.86 (CH_3_) (Figure S1l2). HRMS TOF (ES+) m/z calculated for C_19_H_20_NO_2_Cl_2_ [M + H]^+^ 364.0871, found 364.0862.

#### 3.2.13. Treatment of Compound **7** with 4-Bromobenzyl Bromide in the Presence of NaH: Synthesis of **8c**

To a stirred solution of compound **7** (854 mg, 2.16 mmol) in 30 mL THF, under argon atmosphere, were added 500 mg of NaH (21 mmol). After stirring for 30 min, 1350 mg (5.4 mmol) of 4-bromobenzyl bromide were added portion wise, and the resulting solution was stirred for 8 h at 50 °C. The mixture was then cooled at 0 °C and diluted with 150 mL of MTBE. Then 15 mL of saturated NH_4_Cl were added dropwise. The aqueous layer was extracted with MTBE (3 × 30 mL). The combined organic layers were washed with brine (3 × 40 mL) dried over anhydrous Na_2_SO_4_, and concentrated under reduced pressure. The resulting crude product was dissolved in 50 mL of dry THF and 128 mL of 1M TBAF solution in THF was added to the resulting solution at room temperature and under argon atmosphere. After stirring for 4 h the reaction mixture was diluted with 200 mL of EtOAc, washed with brine (3 × 50 mL), dried over anhydrous Na_2_SO_4_ and concentrated in vacuo. The reaction crude was purified by flash chromatography (H:MTBE, 9:1) to afford 786 mg of compound **8c** (1.75 mmol, 81%). 

**Compound 8c**. ^1^H NMR (600 MHz, CDCl_3_) δ 7.37 (d, *J* = 8.6 Hz, 2H), 7.33 (d, *J* = 8.6 Hz, 2H), 7.33–7.29 (m, 1H), 7.31 (d, *J* = 8.0 Hz, 2H), 7.12 (d, *J* = 7.3 Hz, 1H), 7.08 (dd, *J* = 8.4, 7.7 Hz, 1H), 7.05–7.01 (m, 1H), 6.95–6.90 (m, 1H), 4.86 (s, 2H), 3.55 (t, *J* = 7.0 Hz, 2H), 2.58 (t, *J* = 7.0 Hz, 2H) (Figure S1t). ^13^C NMR (151 MHz, CDCl_3_) δ 145.16 (C), 143.87 (C), 136.74 (C), 135.21 (2C), 131.45 (2CH), 130.57 (C), 130.55 (CH), 130.01 (2CH), 129.35 (2CH), 127.04 (CH), 126.46 (C), 123.21 (CH), 122.68 (CH), 120.68 (C), 62.24 (CH_2_), 55.46 (CH_2_), 34.55 (CH_2_) (Figure S1u). HRMS TOF (ES+) m/z calculated for C_21_H_19_NOCl_2_Br [M + H]^+^ 450.0027, found 450.0021. 

#### 3.2.14. Dess-Martin Oxidation of **8c**

To a stirred solution of **8c** (375 mg, 0.80 mmol) in 25 mL of DCM under argon was added the Dess−Martin reagent (677 mg, 1.6 mmol). After stirring for 50 min at room temperature, the reaction was diluted with DCM (20 mL) and quenched with 10 mL of a saturated solution of Na_2_S_2_O_3_−NaHCO_3_ that was added slowly to the mixture. The organic layer was then washed with brine (3 × 20 mL), dried over anhydrous Na_2_SO_4_, and concentrated under reduced pressure. The crude product was purified by flash chromatography (H:MTBE, 2:1) to obtain 325 mg of **9c** (0.72 mmol, 90%).

**Compound 9c**. ^1^H NMR (600 MHz, CDCl_3_) δ 9.15 (t, *J* = 1.8 Hz, 1H), 7.38 (d, *J* = 8.4 Hz, 2H), 7.31 (d, *J* = 8.1 Hz, 4H), 7.14–7.04 (m, 3H), 7.02 (dd, *J* = 7.6, 1.7 Hz, 1H), 6.96 (td, *J* = 7.3, 1.3 Hz, 1H), 4.82 (s, 2H), 3.44 (d, *J* = 1.8 Hz, 2H) (Figure S1e2). ^13^C NMR (151 MHz, CDCl_3_) δ 199.04 (CHO), 145.66 (C), 143.37 (C), 136.34 (C), 135.37 (2C), 132.22 (CH), 131.51 (2CH), 130.17 (2CH), 129.28 (2CH), 127.62 (CH), 127.60 (CH), 124.93 (C), 123.03 (CH), 122.77 (CH), 120.79 (C), 55.27 (CH_2_), 46.50 (CH_2_) (Figure S1f2). HRMS TOF (ESI+) m/z calculated for C_21_H_17_NOCl_2_Br [M + H]^+^ 447.9871, found 447.9837.

#### 3.2.15. Pinnick Oxidation of **9c**

Aldehyde **9c** (335 mg, 0.72 mmol) was dissolved in 20 mL of *tert*-butyl alcohol and 3 mL of 2-methyl-2-butene. A solution of sodium chlorite (266 mg, 2.94 mmol) and sodium dihydrogenphosphate dihydrate (298 mg, 1.91 mmol) in 5 mL of water was added dropwise over a 20 min period. The yellow reaction mixture was stirred at room temperature for 50 min. Volatile components were then removed under vacuum. The residue was dissolved in 30 mL of water and extracted with 2 50 mL portions of MTBE. The combined organic layers were washed with brine, dried, and concentrated under reduced pressure. Purification by flash chromatography (H:MTBE, 10:1) afforded 131 mg of compound **10c** (0.5 mmol, 69%).

**Compound 10c**: ^1^H NMR (600 MHz, CDCl_3_) δ 7.37 (d, *J* = 8.5 Hz, 2H), 7.32 (d, *J* = 8.4 Hz, 1H), 7.27 (d, *J* = 7.6 Hz, 3H), 7.15–7.06 (m, 2H), 7.03 (d, *J* = 8.1 Hz, 1H), 7.01 (t, *J* = 8.1 Hz, 1H), 6.96 (td, *J* = 7.1, 0.8 Hz, 1H), 4.86 (s, 2H), 3.50 (d, *J* = 4.3 Hz, 2H) (Figure S1l2). ^13^C NMR (101 MHz, CDCl_3_) δ 174.54 (C), 145.26 (C), 143.04 (C), 136.46 (C), 135.53 (2C), 132.06 (CH), 131.46 (2CH), 130.03 (2CH), 129.30 (2CH), 127.56 (CH), 127.33 (CH), 126.03 (C), 123.15 (CH), 122.79 (CH), 120.73 (C), 55.49 (CH_2_), 36.43 (CH_2_) (Figure S1m2). HRMS TOF (ESI+) m/z calculated for C_21_H_17_NO_2_Cl_2_Br [M + H]^+^ 463.9820, found 463.9829.

### 3.3. Cell Viability Assays

All cell lines used in this article were provided by the cell bank of the University of Granada, Spain: HT29 human colorectal adenocarcinoma cell line (ECACC no. 9172201; ATCC no. HTB-38); Hep-G2 human hepatocarcinoma cell line (ECACC no. 85011430; B16-F10 mouse melanoma cell line (ATCC no. CRL-6475), and RAW 264.7 monocyte/macrophage murine cell line (ATCC no. TIB-71). All cell lines were cultured in DMEM supplemented with 2 mM glutamine, 10% heat-inactivated FCS, 50 µg·mL^−1^ of gentamicin, being incubated at 37 °C, in an atmosphere of 5% CO_2_ and 95% humidity. Subconfluent monolayer cells were used in all experiments. 

DCF and its 8 different derivatives were dissolved at 5 mg·mL^−1^ in DMSO, and stored at 4 °C. Before use in cell treatment, these compounds were diluted in cell culture medium at adequate concentrations for each experiment. Apoptosis, mitochondrial-membrane potential and cell-cycle analysis were measured at IC_50_ and IC_80_ in HT29, Hep-G2 and B16-F10. For nitrite concentration, ¾·IC_50_, ½·IC_50_ and ¼·IC_50_ concentrations were used to ensure sub-cytotoxic concentration against RAW 264.7 cells.

The effect of each treatment with compounds **2**, **4**, **6**, **8c**, **9c**, **10a-c**, and DCF on cell cytotoxicity were determined in all cell lines described before. The cells viability was determined by using MTT (3-(4,5-dimethylthiazol-2-yl)-2,5-diphenyltetrazolium bromide) proliferation assay (Sigma, St. Louis, MO, USA), which is based on the ability of live cells to cleave the tetrazolium ring, thus producing formazan, which absorbs at 570 nm. 

For this assay, 6 × 10^3^ HT29 cells, 15 × 10^3^ Hep-G2 cells, 5 × 10^3^ B16-F10 cells and 6 × 10^3^ RAW 264 cells per well were grown on a 96-well plate and incubated with the different products (0–200 μg·mL^−1^). After 72 h, 100 µL of MTT solution (0.5 mg·mL^−1^) in a mixture of 50% of PBS (Phosphate-Buffered Saline) and 50% of cell medium was added to each well. After 1.5 h of incubation, cells were washed twice with PBS, and formazan was resuspended in 100 μL DMSO. Relative cell viability, with respect to untreated control cells, was measured by absorbance at 570 nm on an ELISA plate reader (Tecan Sunrise MR20-301, Männedorf, Switzerland, Männedorf, Switzerland).

### 3.4. Anexin V-FICT/Propidium Iodide Flow-Cytometric Analysis

Apoptosis was analyzed with flow-cytometry by using a FACScan flow-cytometer (fluorescence-activated cell sorter) (Coulter Corporation, Hialeah, FL, USA). In brief, 15 × 10^4^ Hep-G2 cells per well were plated in 24-well plates with 1.5 mL of medium, following treatment with the compounds for 72 h at the IC_50_ and IC_80_ concentrations, calculated previously. The cells were collected and resuspended in binding buffer (10 mM HEPES/NaOH, pH = 7.4, 140 mM NaCl, 2.5 mM CaCl_2_). Annexin V-FITC conjugate (1 mg·mL^−1^) was added and incubated for 30 min at room temperature in darkness. Just before FACS analysis, cells were stained with 20 µL of 1 mg·mL^−1^ propidium iodide (PI) solution. In each experiment, approximately 10 × 10^3^ cells were analyzed, and the experiment was duplicated 3 times. The percentages of apoptosis were determined with annexin V-FICT/PI by FACS (flow-activated cell sorter) cytometric analysis, differentiating early apoptotic cells (An-V^+^ and PI^-^) from late apoptotic (An-V^+^ and PI^+^), necrotic (An-V^-^ and PI^+^) or normal cells (An-V^-^ and PI^-^). 

### 3.5. Cell-Cycle Analysis

The cell-cycle was analyzed again with flow cytometry by using a fluorescence-activated cell sorter (FACS) at 488 nm in an Epics XL flow cytometer (Coulter Corporation, Hialeah, FL, USA). For this assay, 15 × 10^4^ Hep-G2 cells per well were plated in 24-well plates with 1.5 mL of medium, following treatment with the compounds for 72 h at the IC_50_ and IC_80_ concentrations. After treatment, cells were washed twice with PBS and harvested by trypsinization, then were resuspended in 1X TBS (10 Mm Tris, 150 Mm NaCl), followed by the addition of Vindelov Buffer (100 mM Tris, 100 Mm NaCl, 10 mg/mL RNase, 1 mg/mL PI, pH 8). Samples were allowed to stand for 15 min on ice. Just before FACS analysis, cells were stained with 20 µL of 1 mg·mL^−1^ PI solution. DNA content is directly proportional to the PI fluorescence, allowing to determine the percentage of cells in each cell-cycle phase. In this way, we are able to visualize cell subpopulations with differing DNA contents. Changes in DNA concentrations are characteristic of apoptosis and cell-cycle arrest. The data were analyzed to determine the percentage of cells at each phase of the cell cycle (G0/G1, S, and G2/M). 

### 3.6. Flow-Cytometry Analysis of the Mitochondrial-Membrane Potential

The loss of mitochondrial membrane potential (MMP) during the apoptotic process has been related to the intrinsic mechanism of apoptosis activation. To approach the mechanism of apoptosis implicated in the apoptotic response of Hep-G2 cells, we analyzed the MMP with rhodamine 123 (Rh123) stained. This compound is a membrane-permeable fluorescent cationic dye selectively taken up by mitochondrial, and its fluorescence is proportional to the mitochondrial membrane potential. Oxidative damage was studied by flow-cytometry analysis of the mitochondrial membrane potential, using dihydrorhodamine 123 (DHR) oxidized to the highly fluorescent product rhodamine 123 (Rh123). The formation of Rh123 can be monitored by fluorescence spectroscopy using excitation and emission wavelengths at 500 and 536 nm, respectively. The intracellular measurement of the mitochondrial membrane potential was made by cytometry determination of Rh123. In the same way as in the apoptosis assays, 15 × 10^4^ Hep-G2 cells per well were plated in 24-well plates with 1.5 mL of medium, following treatment with the compounds for 72 h at the IC_50_ and IC_80_ concentrations. After treatment, the medium was removed and a fresh medium with DHR was added at a final concentration of 5 µg/mL. After 30 min of incubation, the medium was removed, and the cells were washed and resuspended in PBS with 5 µg·mL^−1^ of PI. The intensity of fluorescence from Rh123 and PI was determined using a FACScan flow-cytometer (Coulter Corporation, Hialeah, FL, USA). 

### 3.7. Determination of Nitrite Concentration

The concentration of nitrites was determined in assay based on the Griess reaction. This nitrite concentration was used as an indicator of NO production. RAW 264.7 cells were plated at 6 × 10^4^ cells/well in 24-well cell culture plates, supplemented with 10 µg/mL of LPS, except untreated negative control cells. After 24 h of plating, the cells were incubated for 24 h with the test compounds at ¾·IC_50_, ½·IC_50_, and ¼·IC_50_ concentrations determined by MTT proliferation assay. The supernatants were collected at 24, 48, and 72 h to determine nitrite concentration and/or stored at −80 °C for further use. 

For the Griess reaction, 150 µL of supernatant test samples or sodium nitrite standard (0–120 µM) were taken and mixed with 25 µL of the Griess reagent A (0.1% *N*-*N*-(1-naphthyl)-ethylenediamine dihydrochloride) and 25 µL of the Griess reagent B (1% sulfanilamide in 5% of phosphoric acid), in a 96-well plate. After 15 min of incubation at room temperature, the absorbance was measure at 540 nm on an ELISA plate reader (Tecan Sunrise MR20-301, Männedorf, Switzerland, Männedorf, Switzerland). The absorbance was referred to nitrite standard curve to determine the concentration of nitrite in the supernatant. The percentage of NO production was determined, assigning 100% at the increase between negative control (untreated cells) and positive control (cells only treated with 10 µg·mL^−1^ of LPS).

### 3.8. Statistics

Statistical and non-linear sigmoidal regression analyses were performed with the SigmaPlot 12.5 software (Systat Softwre Inc. San Jose, CA, USA). All quantitative data were summarized as the means ± standard deviation (SD). All data shown here were representative of at least 2 independent experiments performed in triplicate.

## Figures and Tables

**Table 1:** Growth-inhibitory effects as IC_20_, IC_50_, and IC_80_ (µg·mL^−1^) values of compounds **2**, **4**, **6**, **8c**, **9c**, **10a**-**c,** and DCF on the three cancer cell lines B16-F10, Hep-G2, and HT29. The last column represents the ratio between IC_50_ values of DCF and the corresponding N-derivative.

Cell Line	Compound #	IC_20_	IC_50_	IC_80_	IC_50_ of DCF/ IC_50_ of Compound
**B16-F10**	**DCF**	34.58 ± 8.46	52.45 ± 3.58	83.75 ± 0.54	1.0
	**2**	21.94 ± 3.44	40.77 ± 1.33	56.59 ± 1.29	1.3
	**4**	37.15 ± 0.07	37.79 ± 0.17	N/A	1.4
	**6**	28.22 ± 2.82	45.77 ± 6.46	N/A	1.1
	**8c**	14.83 ± 3.24	25.55 ± 1.19	42.01 ± 1.59	2.1
	**9c**	21.19 ± 2.00	27.35 ± 0.14	33.59 ± 1.06	1.9
	**10a**	26.79 ± 5.11	N/A	N/A	N/A
	**10b**	6.93 ± 5.48	35.45 ± 4.30	80.98 ± 0.33	1.5
	**10c**	27.42 ± 6.76	48.20 ± 3.05	63.56 ± 1.19	1.1
					
**Hep-G2**	**DCF**	32.48 ± 2.51	46.87 ± 0.97	76.87 ± 1.60	1.0
	**2**	18.45 ± 0.56	24.53 ± 1.11	35.28 ± 3.91	1.9
	**4**	7.53 ± 3.02	14.56 ± 3.07	33.06 ± 2.07	3.2
	**6**	12.30 ± 1.00	21.58 ± 1.77	52.70 ± 7.38	2.2
	**8c**	12.83 ± 1.12	18.48 ± 1.25	29.74 ± 3.03	2.5
	**9c**	17.62 ± 1.42	28.60 ± 1.75	49.21 ± 4.22	1.6
	**10a**	23.15 ± 8.17	78.93 ± 10.67	146.47 ± 13.76	0.6
	**10b**	40.63 ± 9.57	111.26 ± 16.58	184.08 ± 27.89	0.4
	**10c**	17.41 ± 1.08	21.89 ± 0.90	32.33 ± 3.34	2.1
					
**HT29**	**DCF**	45.27 ± 0.52	52.63 ± 0.54	65.90 ± 2.12	1.0
	**2**	18.56 ± 1.26	28.66 ± 1.91	49.14 ± 6.00	1.8
	**4**	3.90 ± 4.74	19.80 ± 9.02	173.90 ± 25.19	2.7
	**6**	7.70 ± 4.60	13.23 ± 4.24	30.53 ± 11.87	4.0
	**8c**	14.10 ± 1.37	20.65 ± 0.76	30.48 ± 0.66	2.5
	**9c**	17.94 ± 1.06	24.93 ± 1.21	41.02 ± 3.24	2.1
	**10a**	62.79 ± 15.01	95.77 ± 7.88	113.61 ± 5.75	0.5
	**10b**	48.12 ± 2.33	69.52 ± 2.86	96.54 ± 1.50	0.8
	**10c**	31.10 ± 3.38	69.30 ± 2.36	103.99 ± 0.79	0.8

**Table 2:** Growth-inhibitory effects as IC_20_, IC_50_, and IC_80_ (µg·mL^−1^) values of compounds **2**, **4**, **6**, **8c**, **9c**, **10a-c,** and DCF against RAW 264.7 monocyte/macrophage murine cells. The last column represents the ratio between IC_50_ of DCF and the IC_50_ of the derivatives.

Cell line	Comp.	IC_20_	IC_50_	IC_80_	IC_50_ of DCF/ IC_50_ of Comp.
**RAW 264.7**	**DCF**	51.91 ± 0.89	69.00 ± 1.13	90.52 ± 1.85	1.0
	**2**	26.43 ± 3.68	30.99 ± 0.77	36.36 ± 3.44	2.2
	**4**	13.87 ± 0.89	17.90 ± 1.31	23.29 ± 2.41	3.9
	**6**	14.68 ± 1.30	19.08 ± 1.47	25.21 ± 2.27	3.6
	**8c**	14.01 ± 0.14	16.71 ± 0.47	20.07 ± 1.27	4.1
	**9c**	20.30 ± 0.83	22.70 ± 1.03	25.50 ± 1.51	3.0
	**10a**	41.42 ± 7.87	72.92 ± 3.57	95.37 ± 1.23	0.9
	**10b**	28.56 ± 1.10	35.64 ± 4.83	45.64 ± 12.53	1.9
	**10c**	35.29 ± 2.83	47.17 ± 3.66	64.92 ± 6.97	1.5

## Data Availability

Data availability upon request.

## Supplementary Materials

The following are available online at https://www.mdpi.com/article/10.3390/ijms22105067/s1, Figure S1. copies of NMR spectra of the synthesized compounds.
