# Supplementary material for: Diclofenac N-Derivatives as Therapeutic Agents with Anti-Inflammatory and Anti-Cancer Effect
Source: Int J Mol Sci. 2021 May 11;22(10):5067. doi: 10.3390/ijms22105067 (PMC8151993; doi:10.3390/ijms22105067)
Supplement: Supplementary file 1 [file ijms-22-05067-s001.zip › ijms-1215330-supplementary.pdf]

Supporting information

Diclofenac N-Derivatives as Therapeutic Agents with Anti-Inflammatory and Anti-Cancer Effect

Alberto Galisteo, Fatin Jannus, Amalia García-García, Houssam Aheget, Sara Rojas, José A. Lupiañez, Antonio Rodríguez-Diéguez, Fernando J. Reyes-Zurita * and José F. Quílez del Moral *

Table of Content

Figure S1. Copies of NMR spectra of the synthesized spectra S2-S27

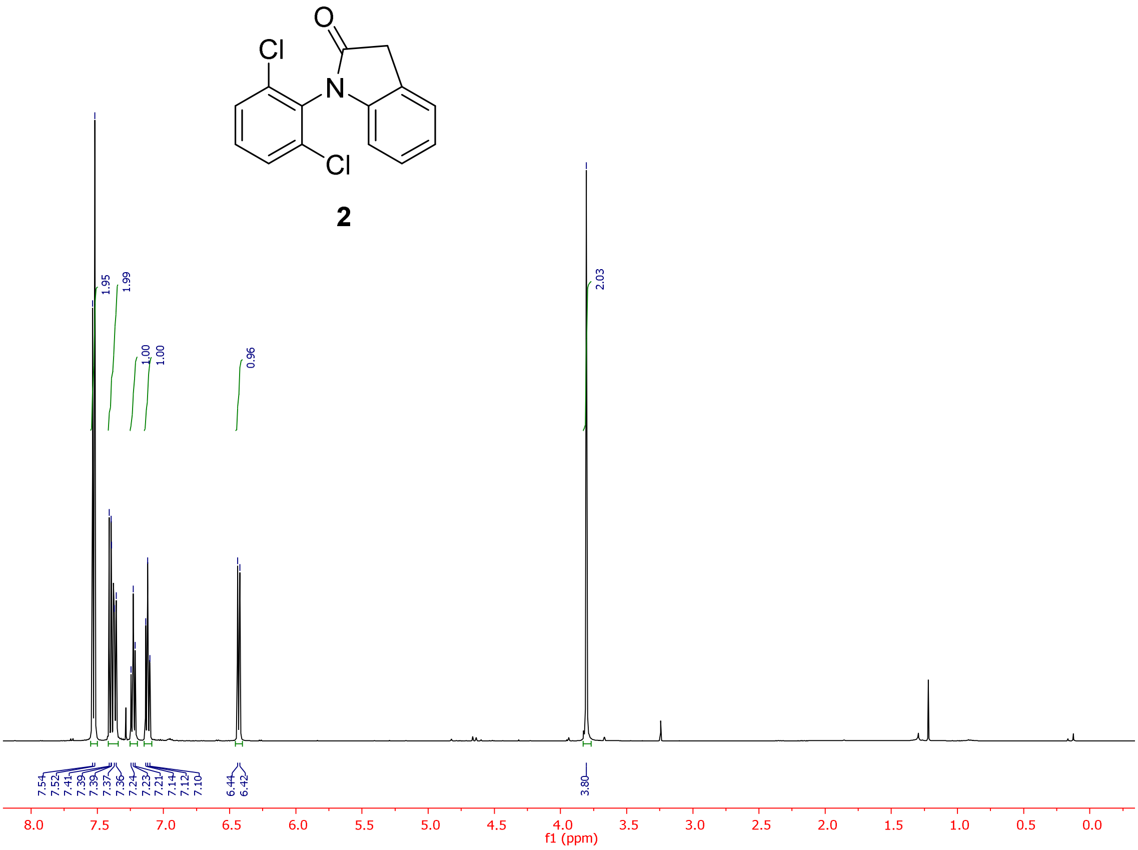

Figure S1a. ^{1}H NMR spectrum of compound **2**

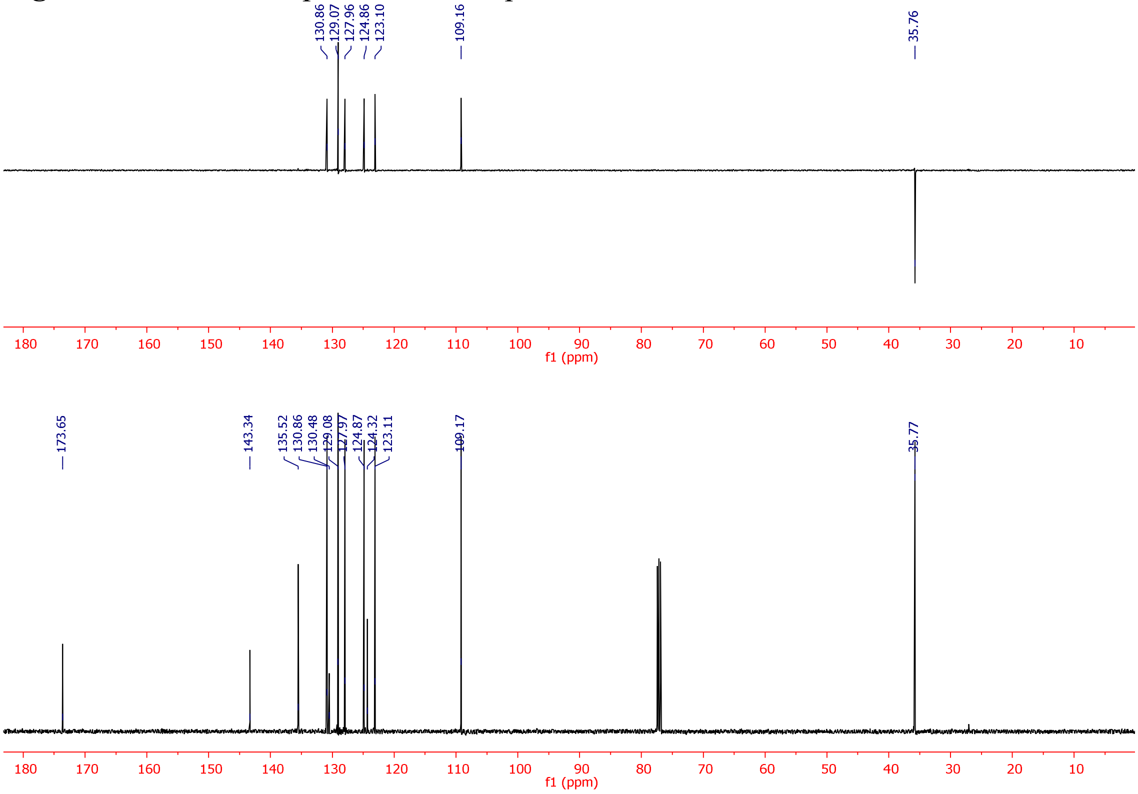

Figure S1b. ^{13}C NMR spectrum of compound **2**

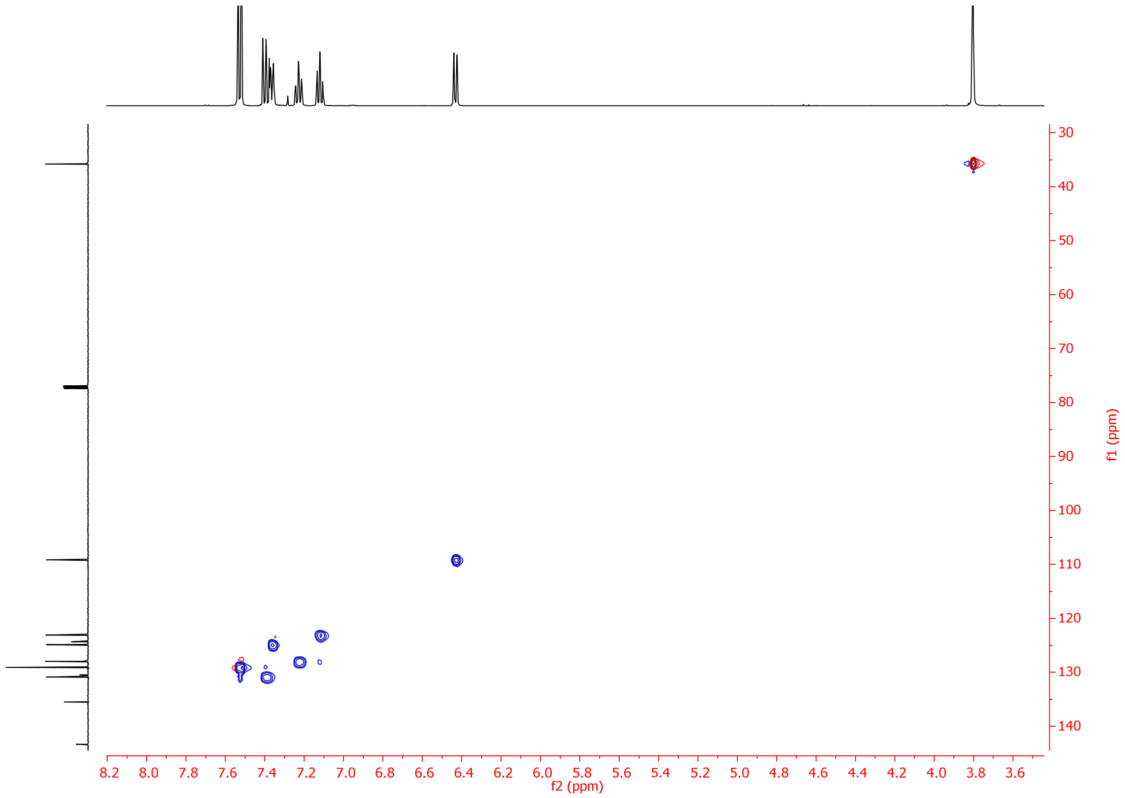

Figure S1c. HSQC of compound **2**

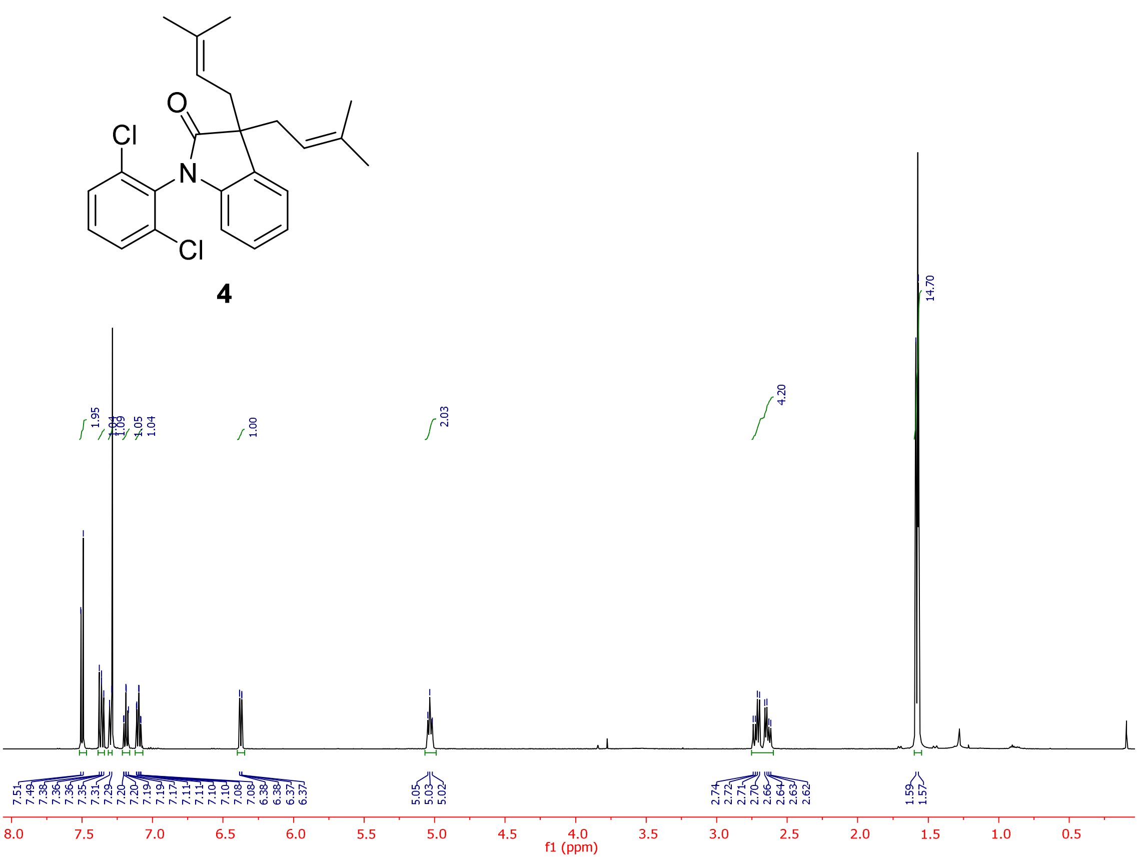

Figure S1d. ^{1}H NMR spectrum of compound **4**

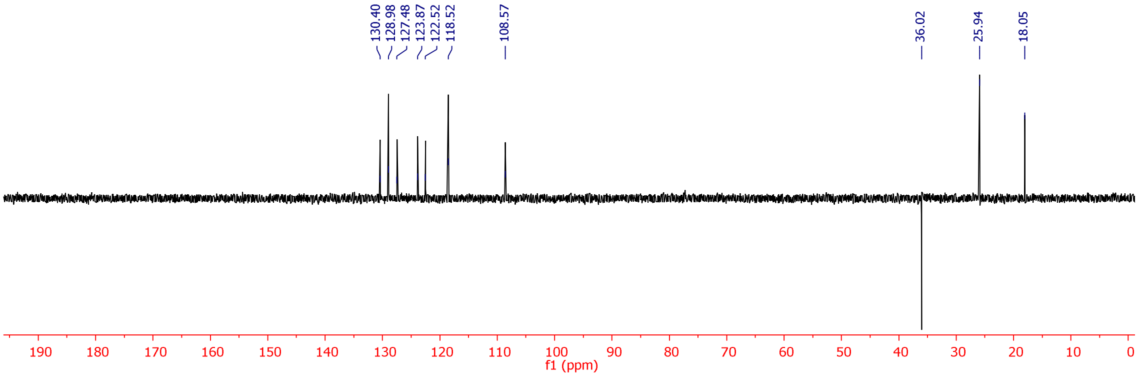

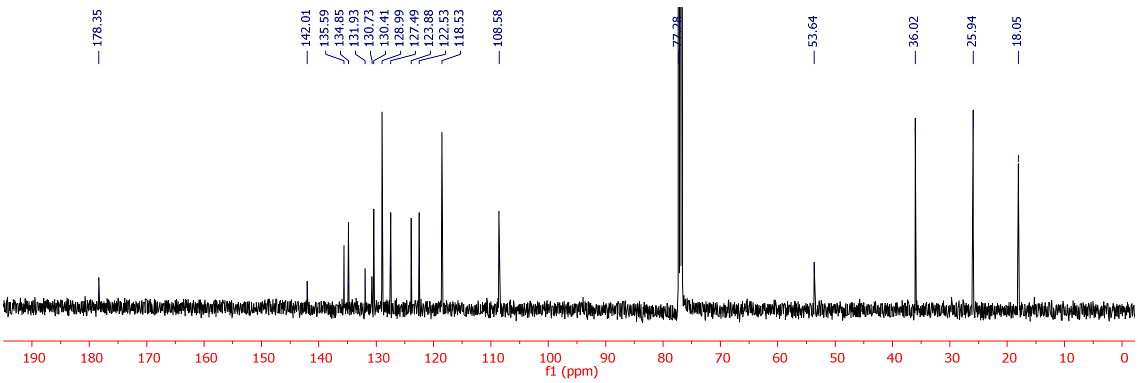

Figure S1e. ^{13}C NMR spectrum of compound **4**

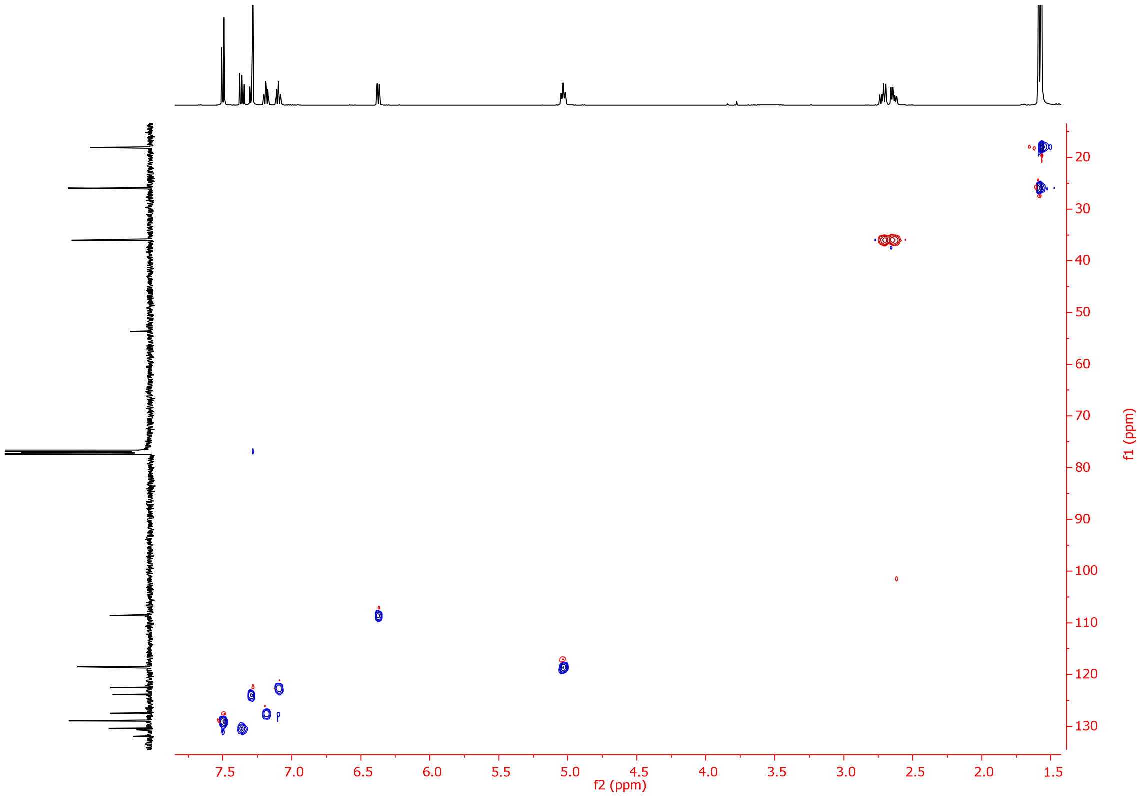

Figure S1f. HSQC of compound **4**

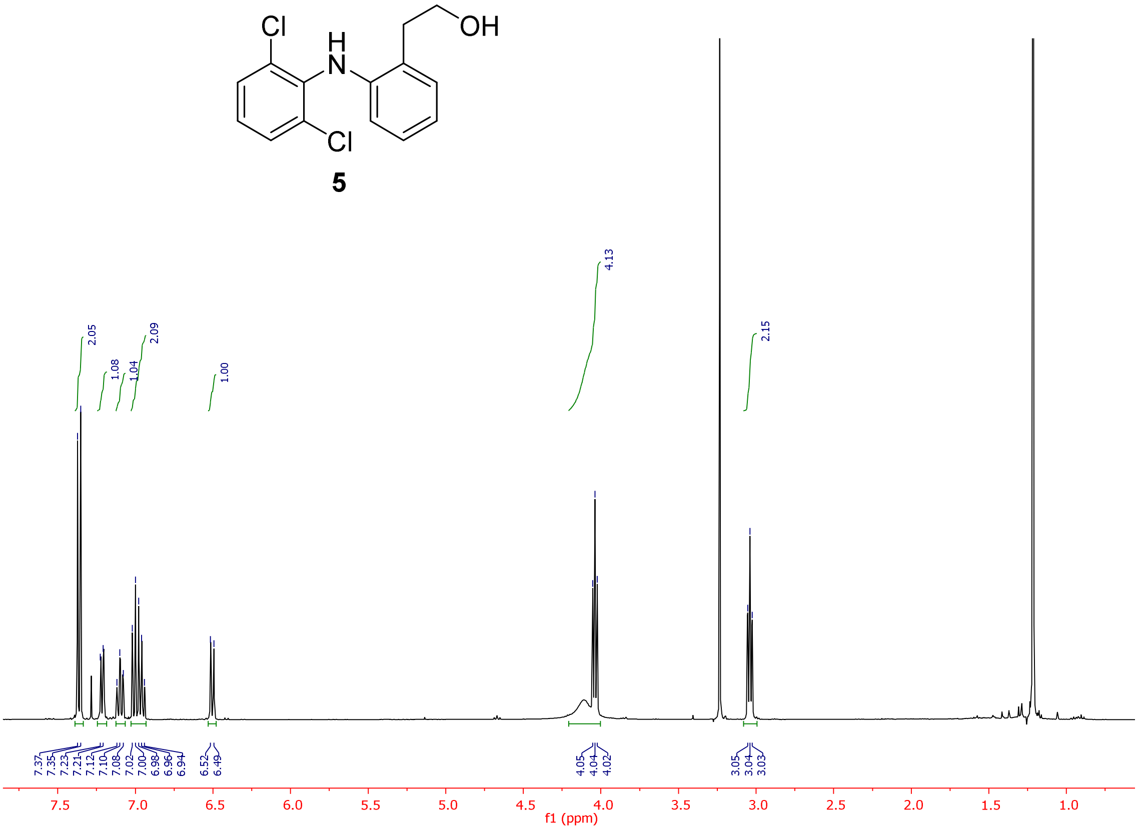

Figure S1g. ^{1}H NMR spectrum of compound **5**

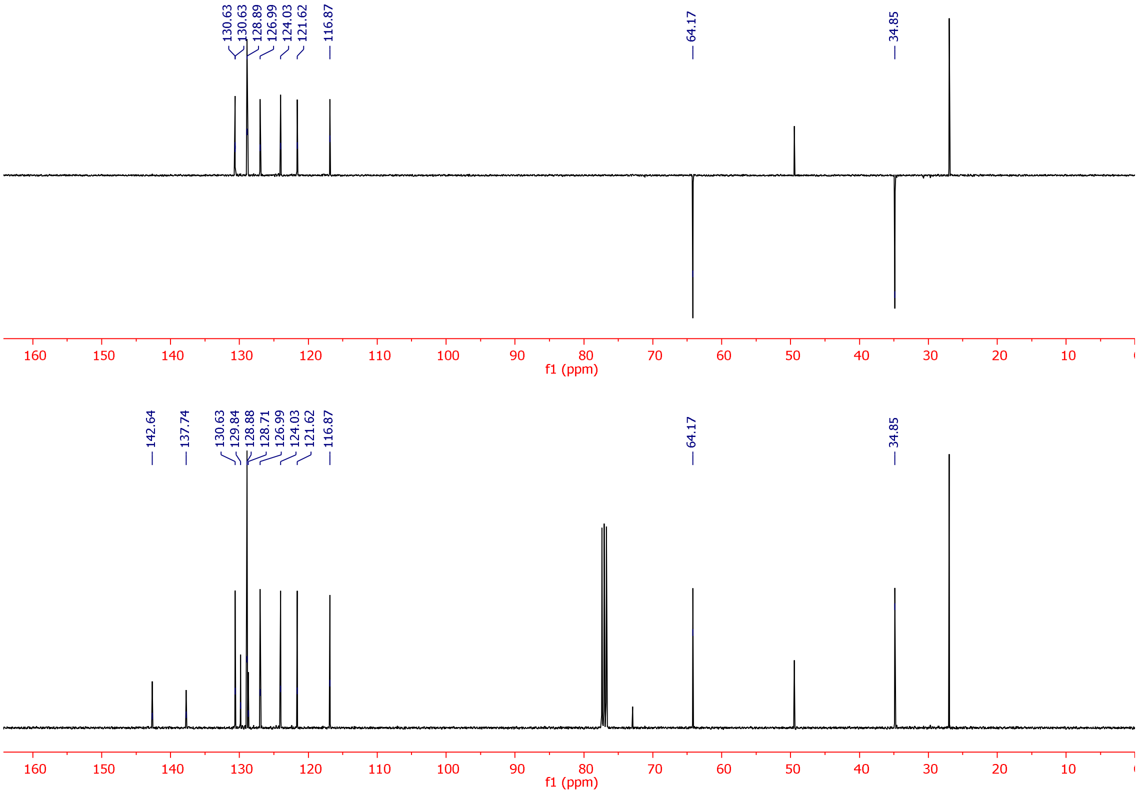

Figure S1h. ^{13}C NMR spectrum of compound **5**

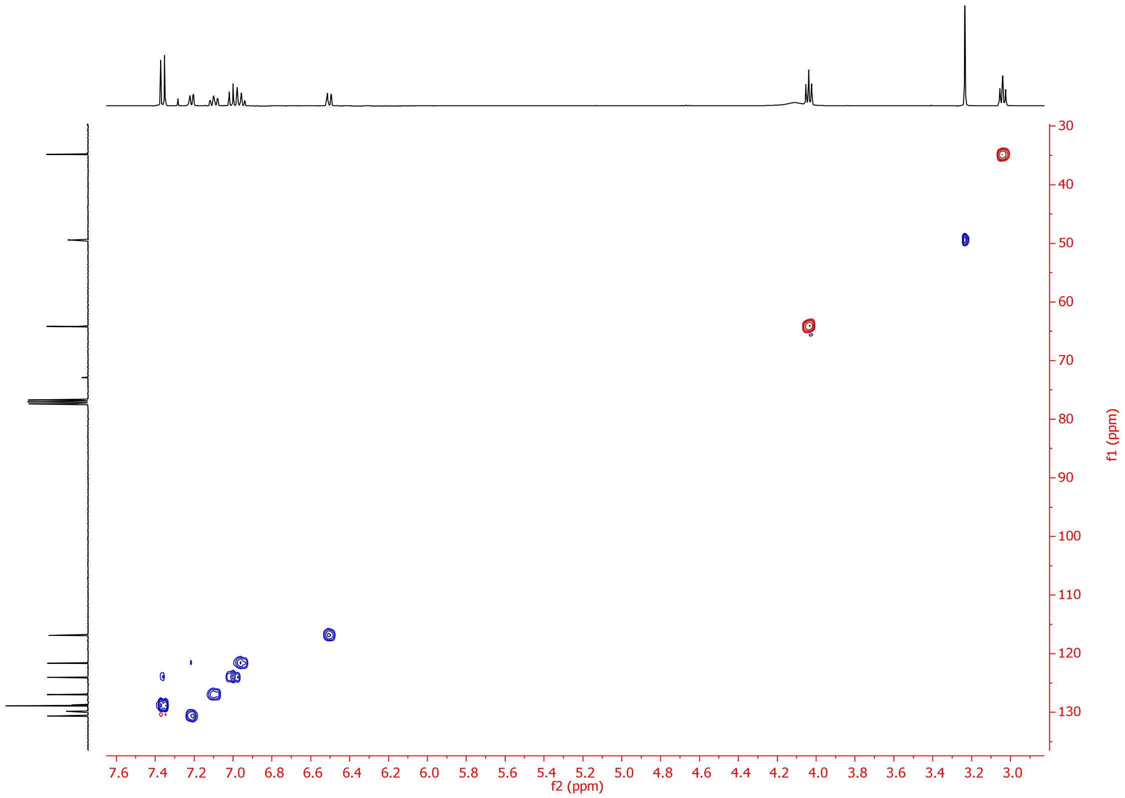

Figure S1i. HSQC of compound **5**

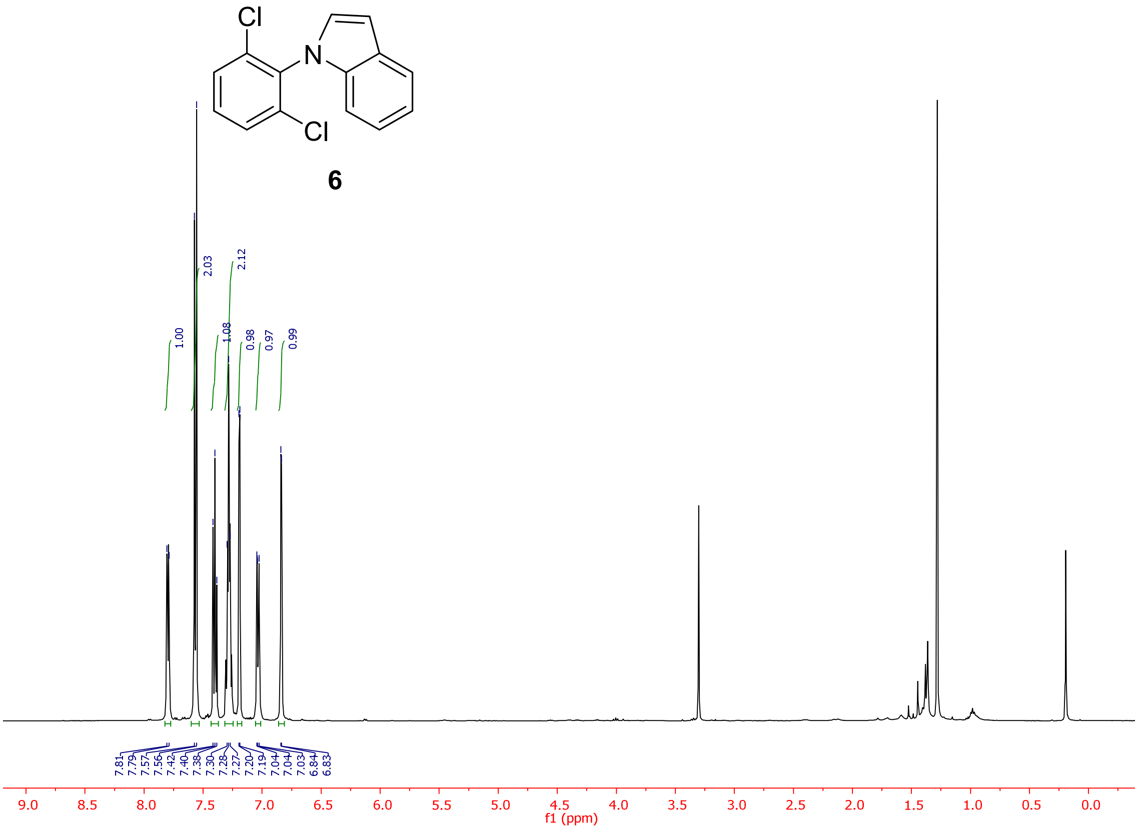

Figure S1j. ^{1}H NMR spectrum of compound **6**

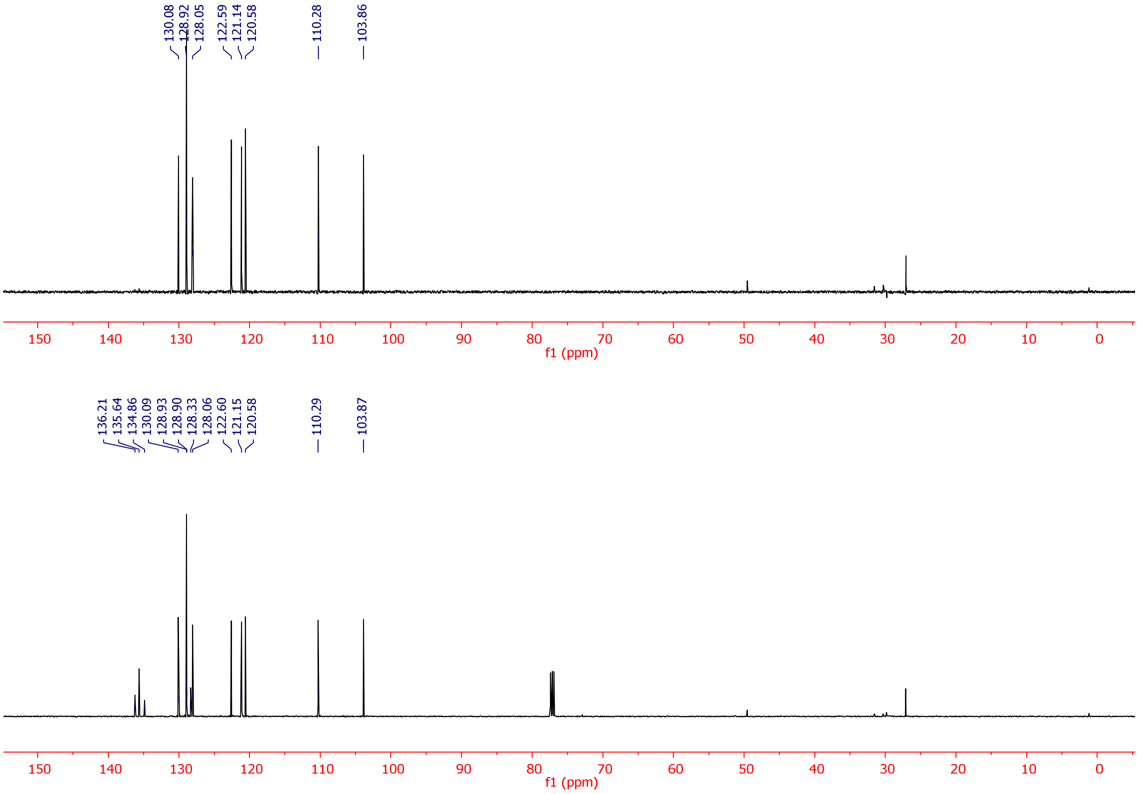

Figure S1k. ^{13}C NMR spectrum of compound **6**

7

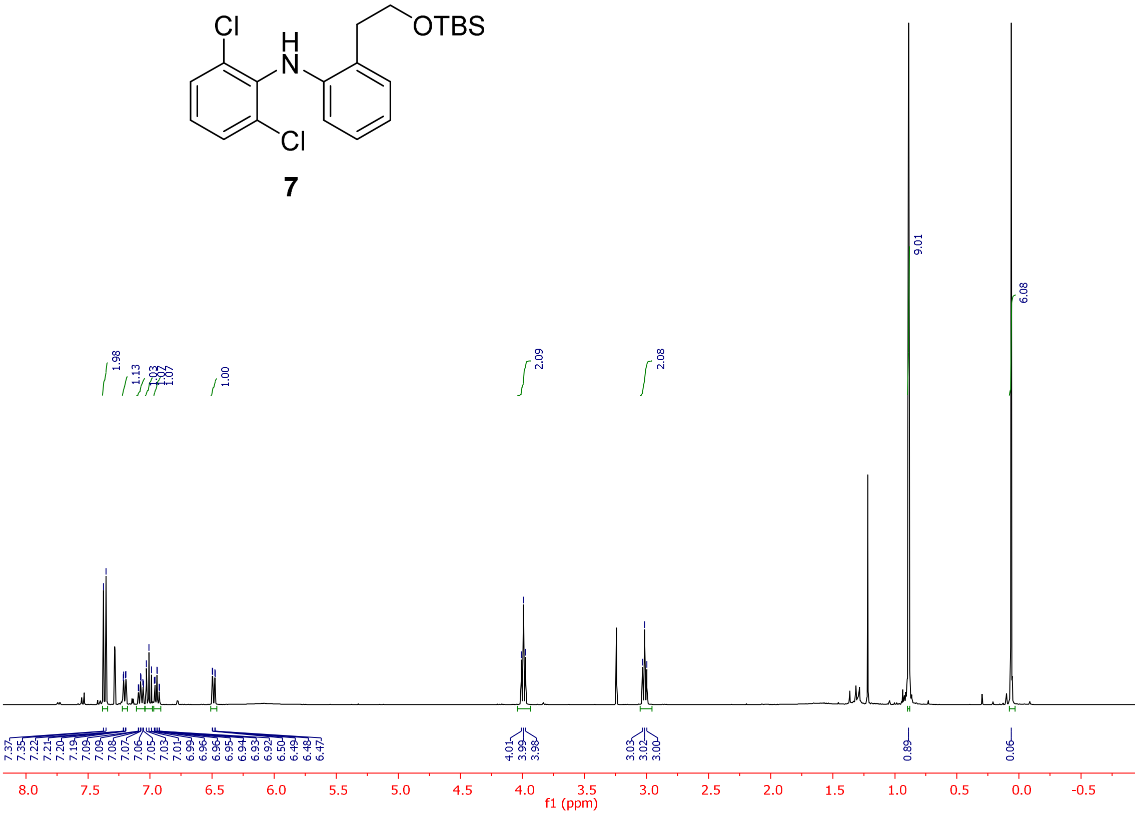

Figure S1l. ^{1}H NMR spectrum of compound **7**

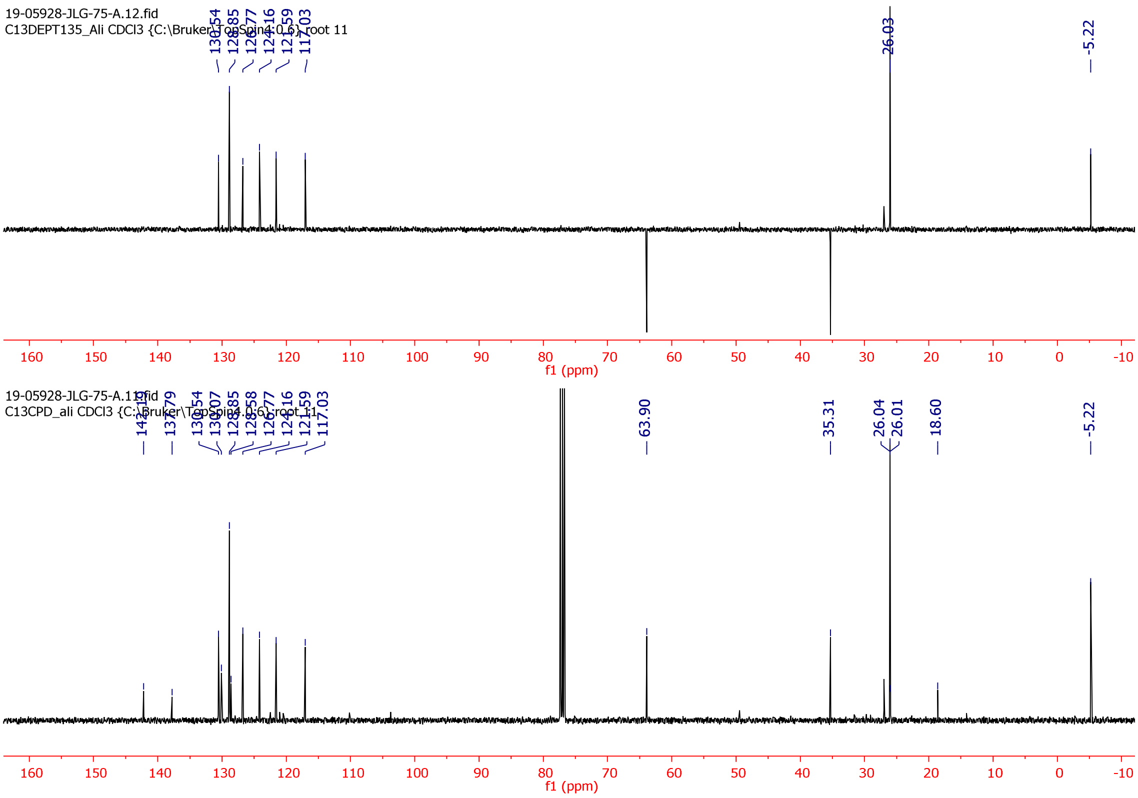

Figure S1m. ^{13}C NMR spectrum of compound **7**

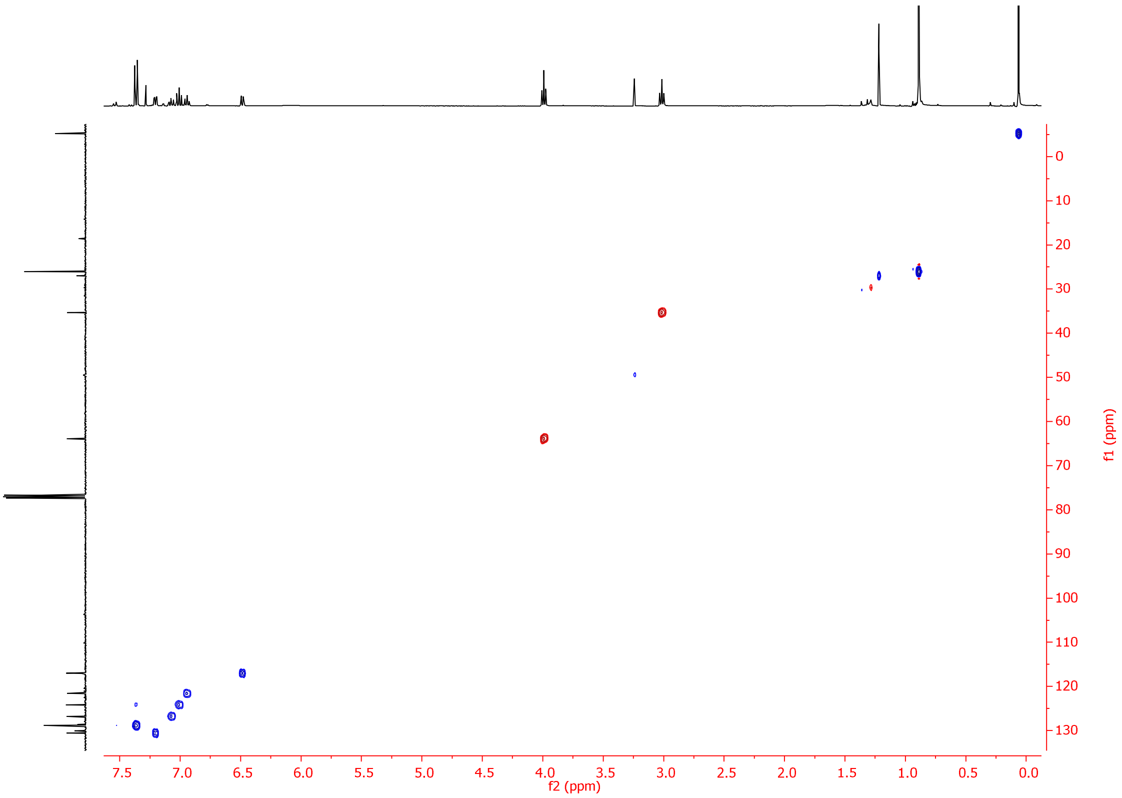

Figure S1n. HSQC of compound **7**

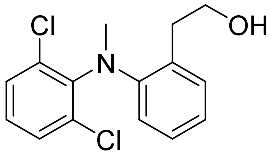

8a

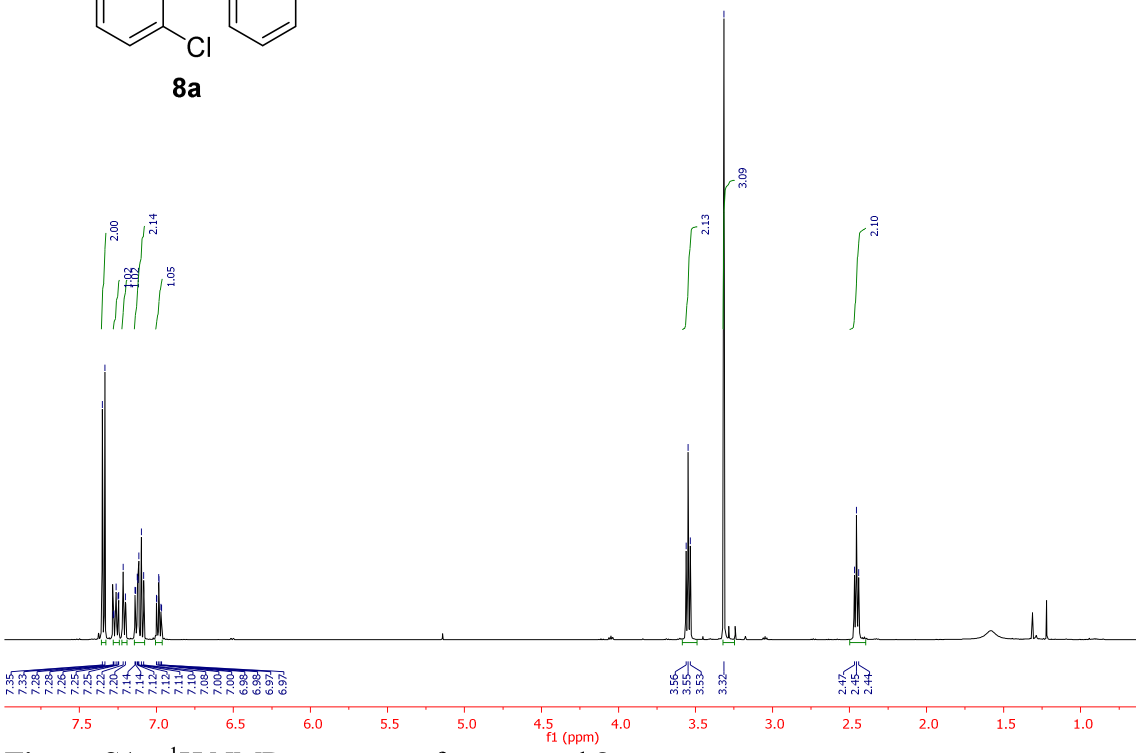

Figure S1o. ^{1}H NMR spectrum of compound **8a**

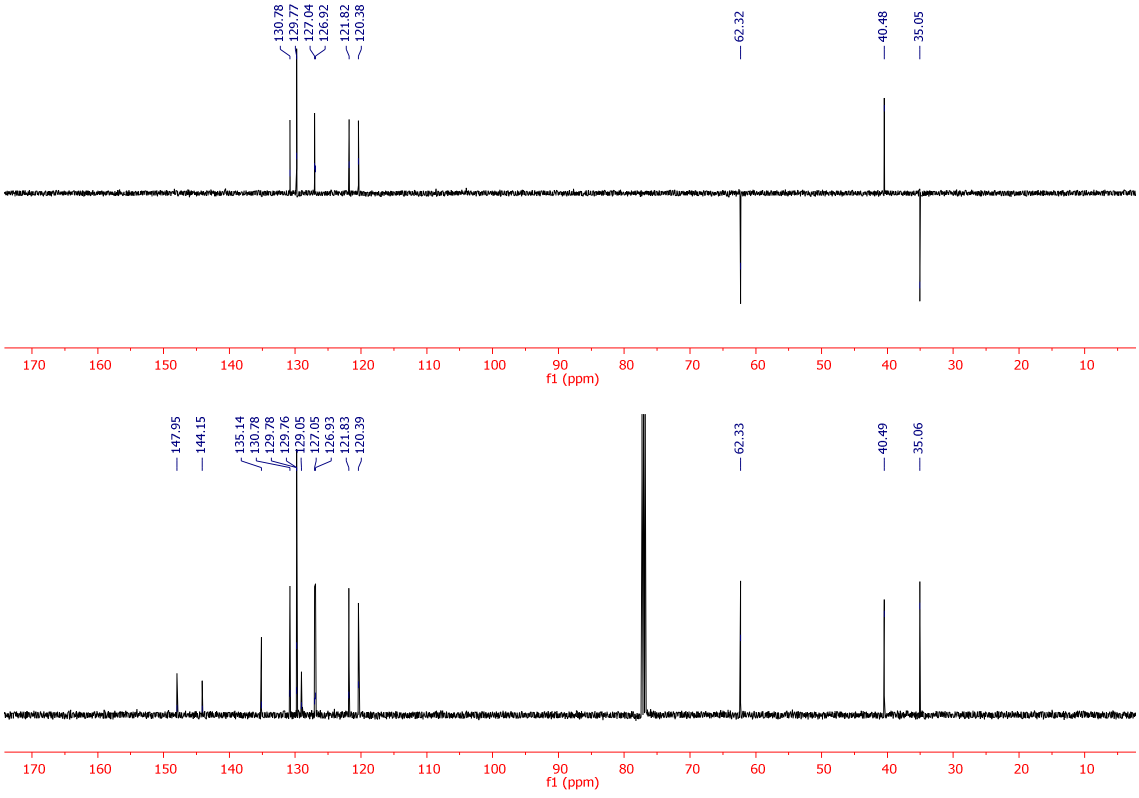

Figure S1p. ^{13}C NMR spectrum of compound **8a**

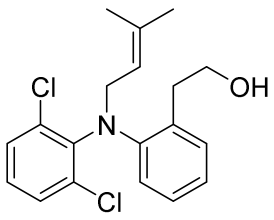

8b

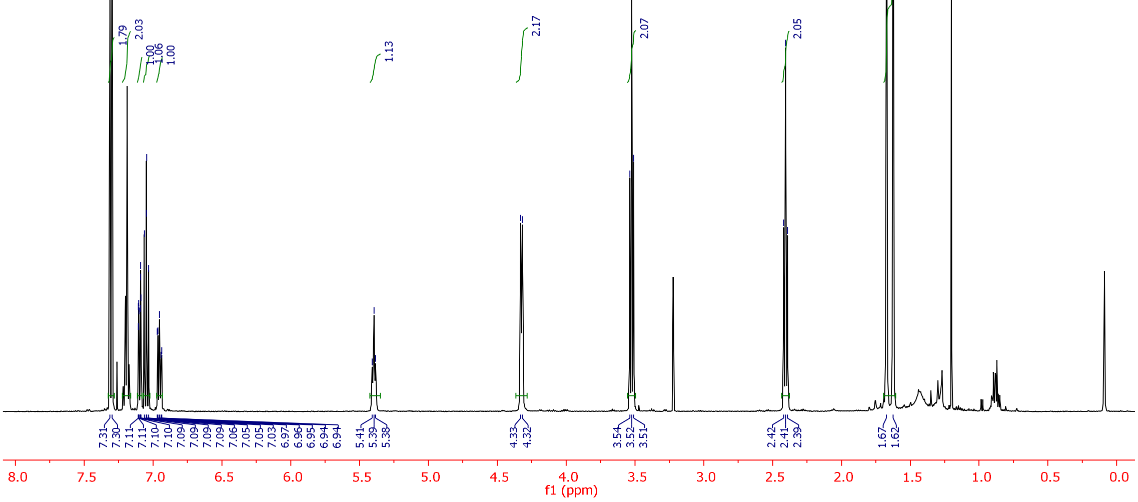

Figure S1q. ^{1}H NMR spectrum of compound **8b**

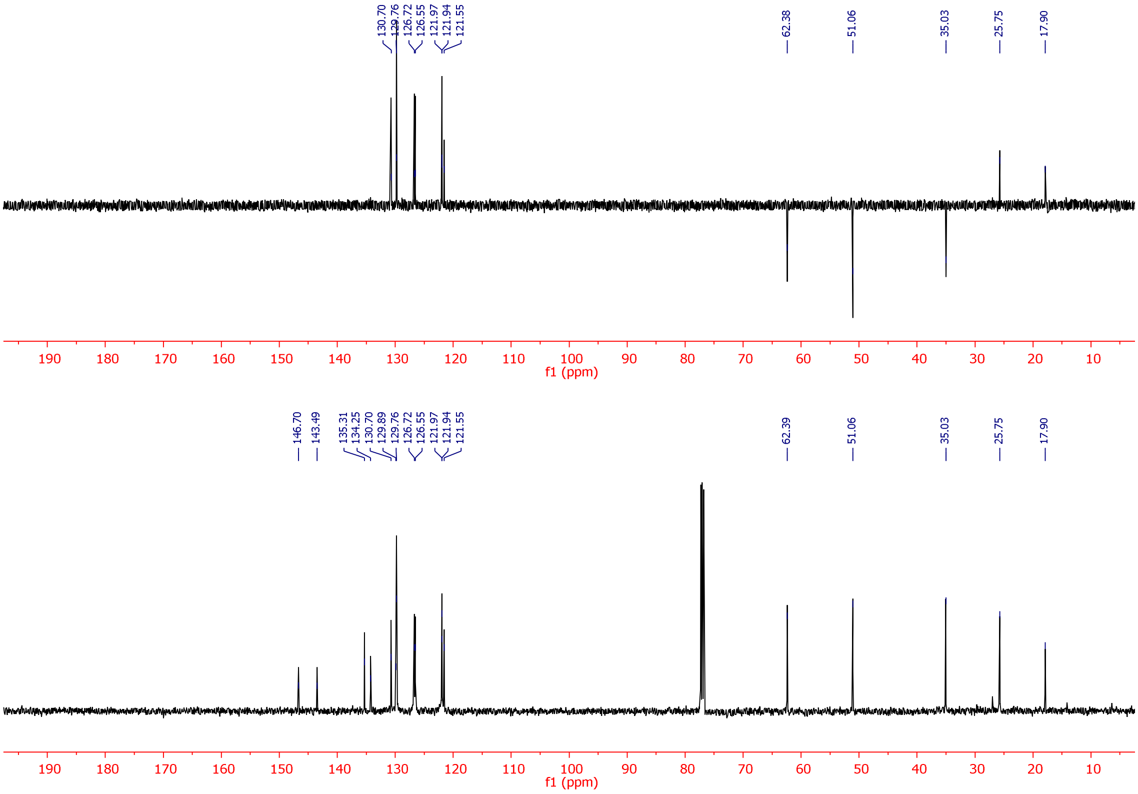

Figure S1r. ^{13}C NMR spectrum of compound **8b**

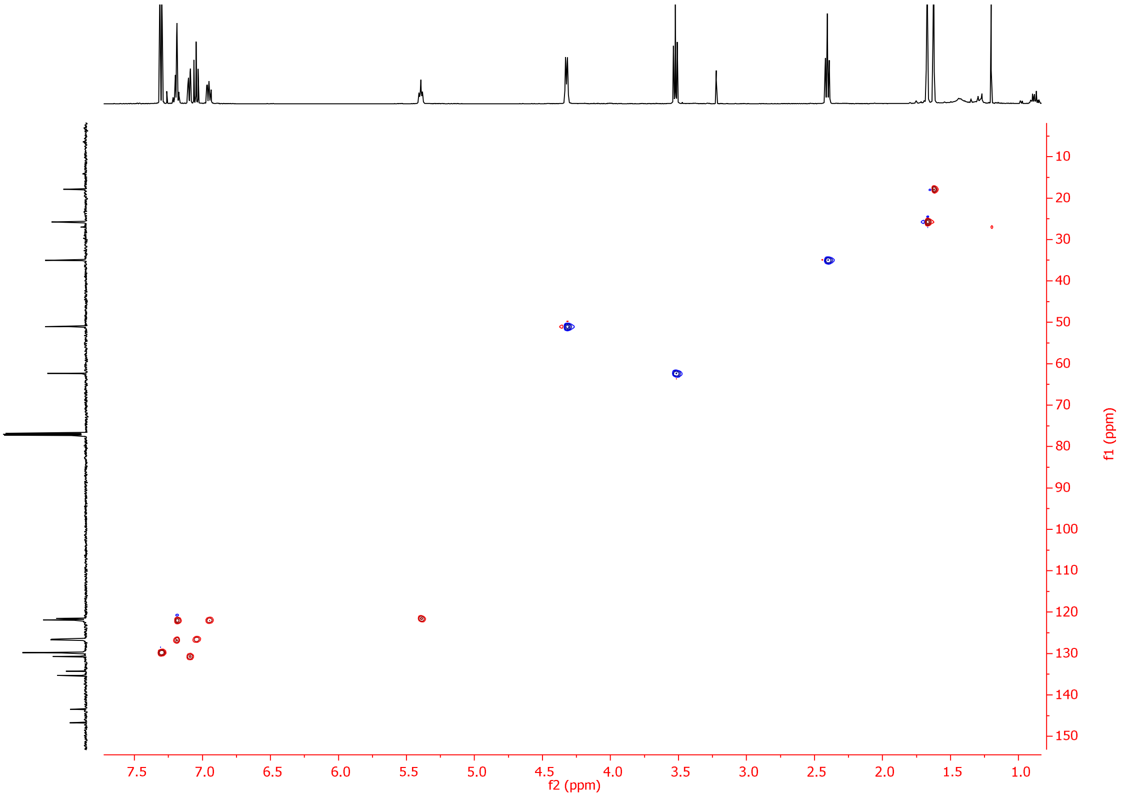

Figure S1s. HSQC of compound **8b**

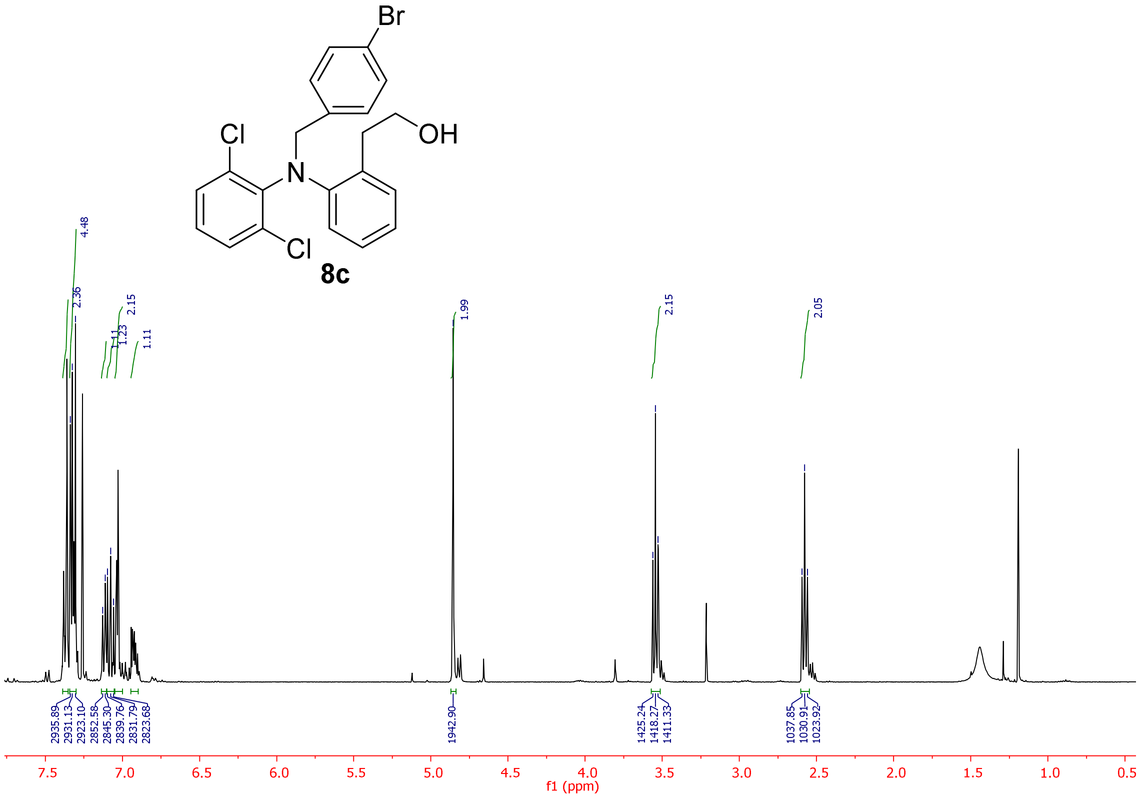

Figure S1t. ^{1}H NMR spectrum of compound **8c**

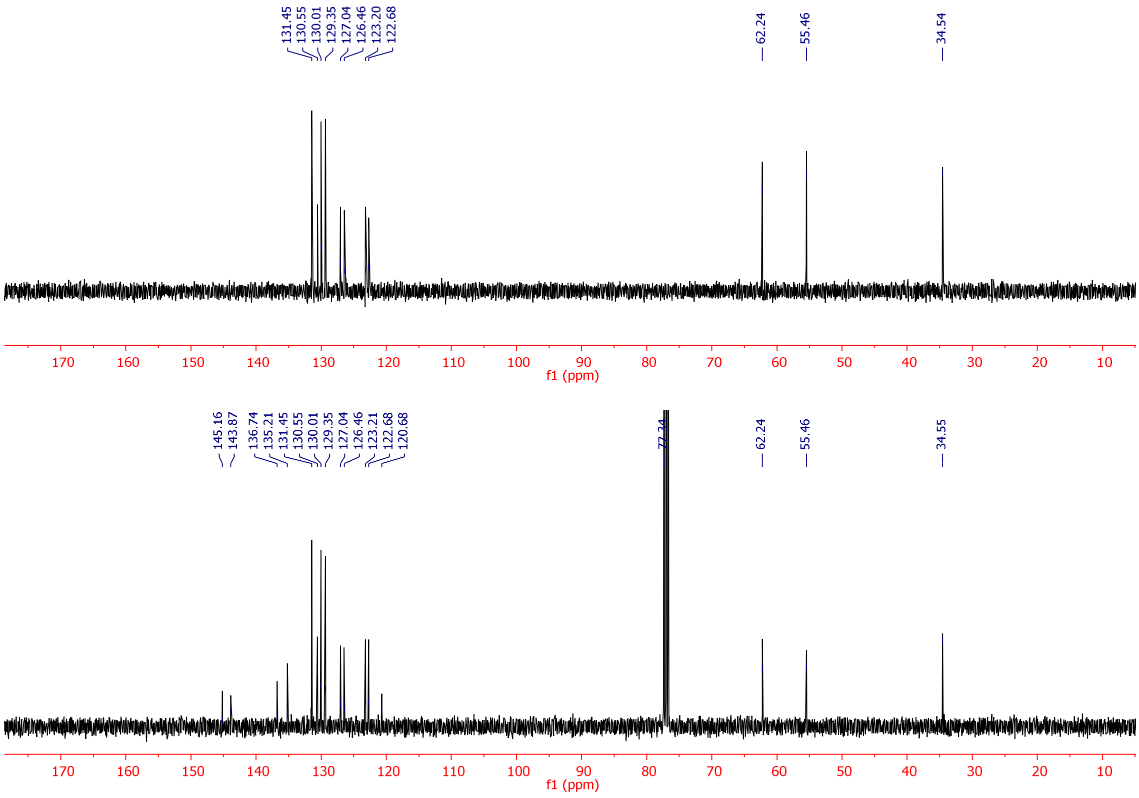

Figure S1u. ^{13}C NMR spectrum of compound **8c**

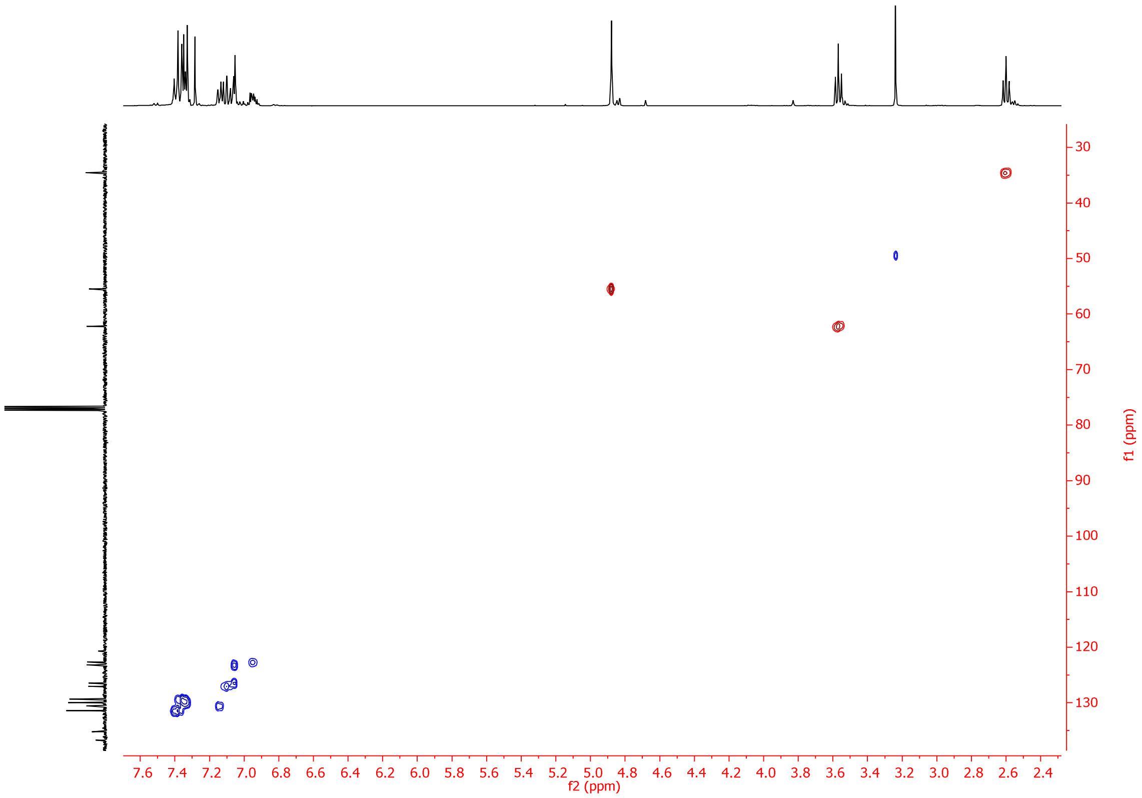

Figure S1w. HSQC of compound **8c**

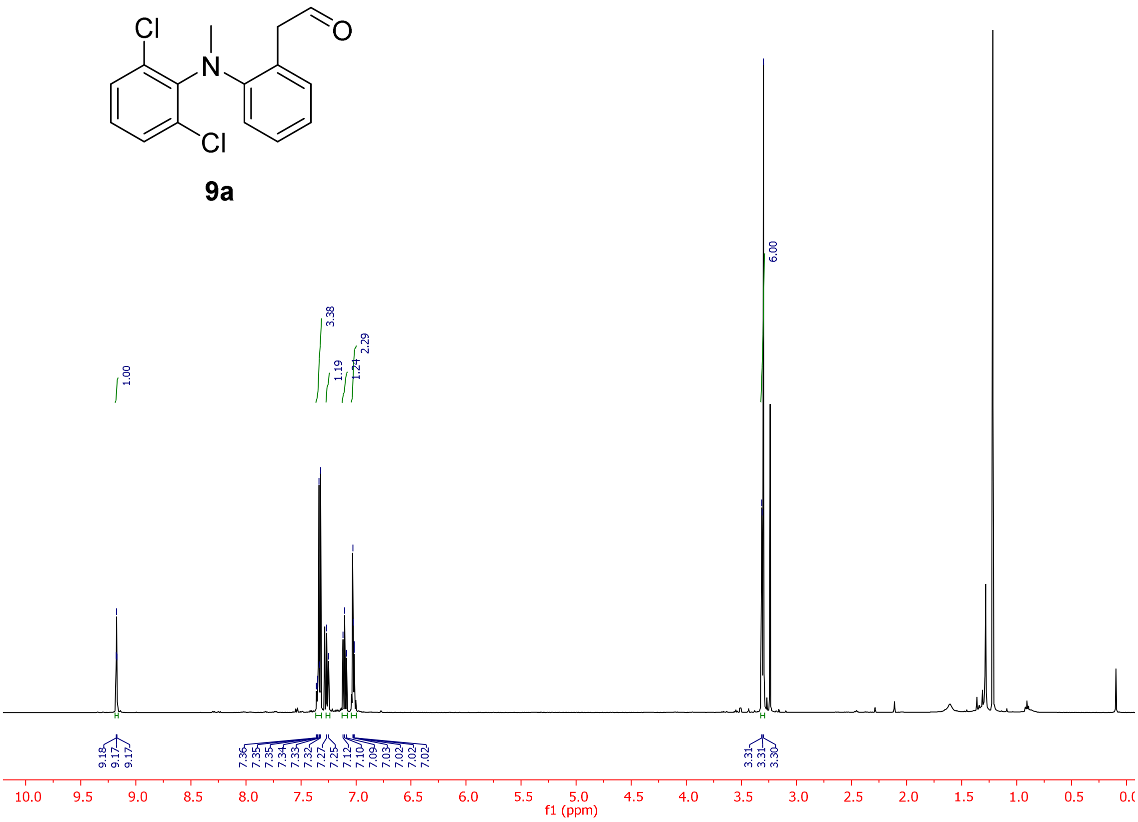

Figure S1y. ¹H NMR spectrum of compound **9a**

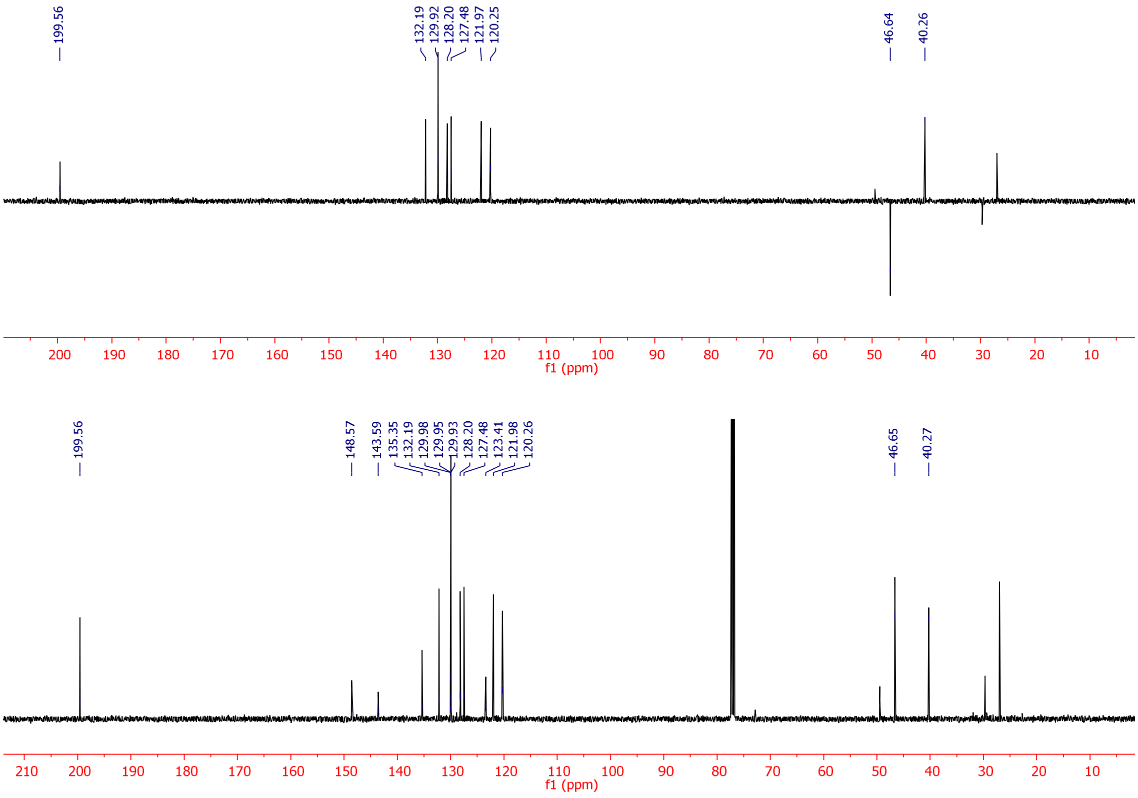

Figure S1z. ^{13}C NMR spectrum of compound **9a**

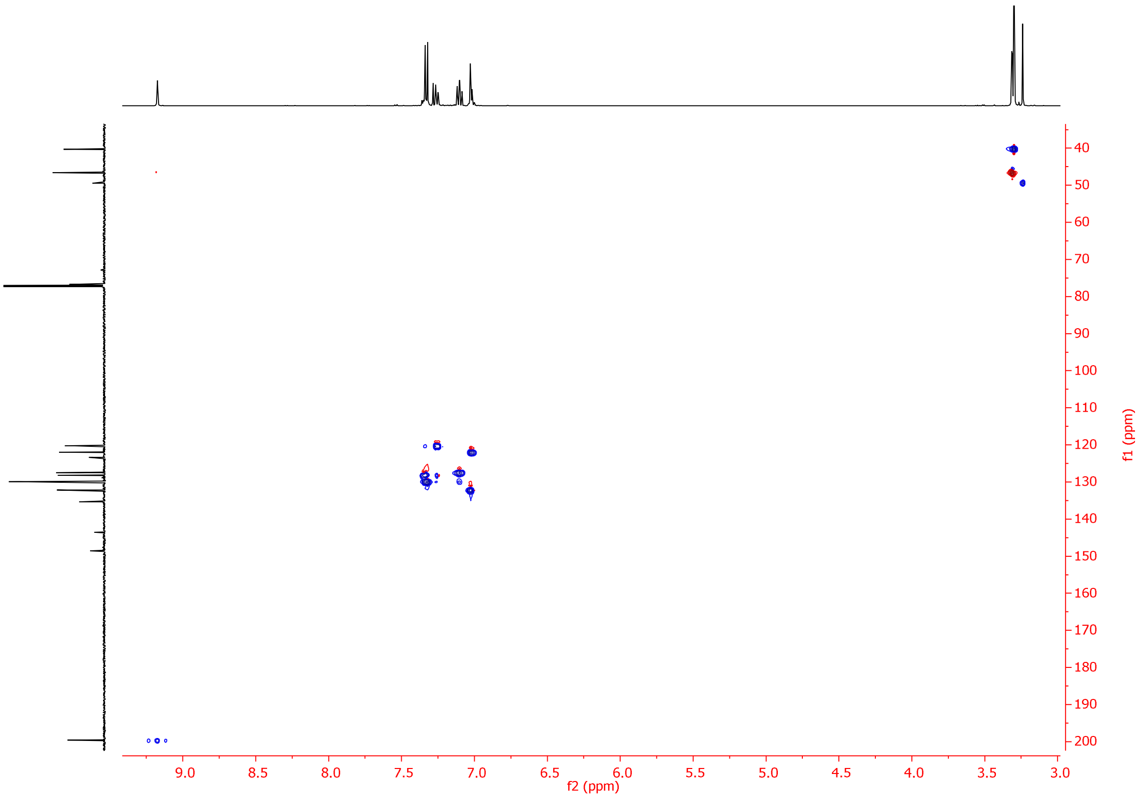

Figure S1a2. HSQC of compound **9a**

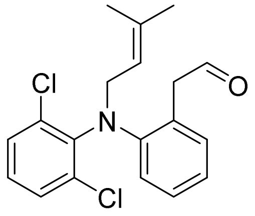

9b

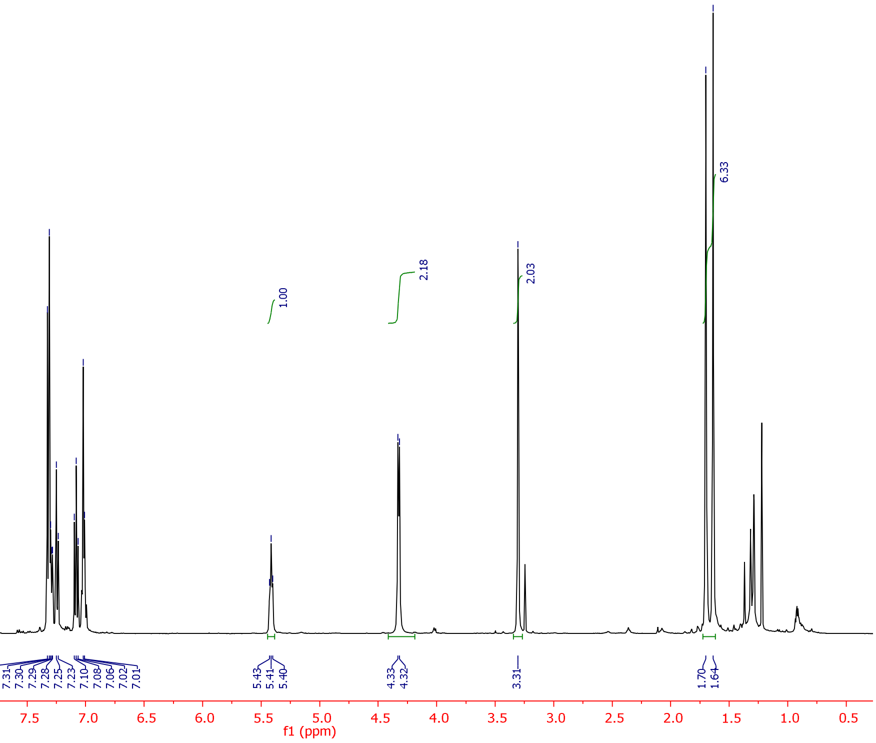

Figure S1b2. ^{1}H NMR spectrum of compound **9b**

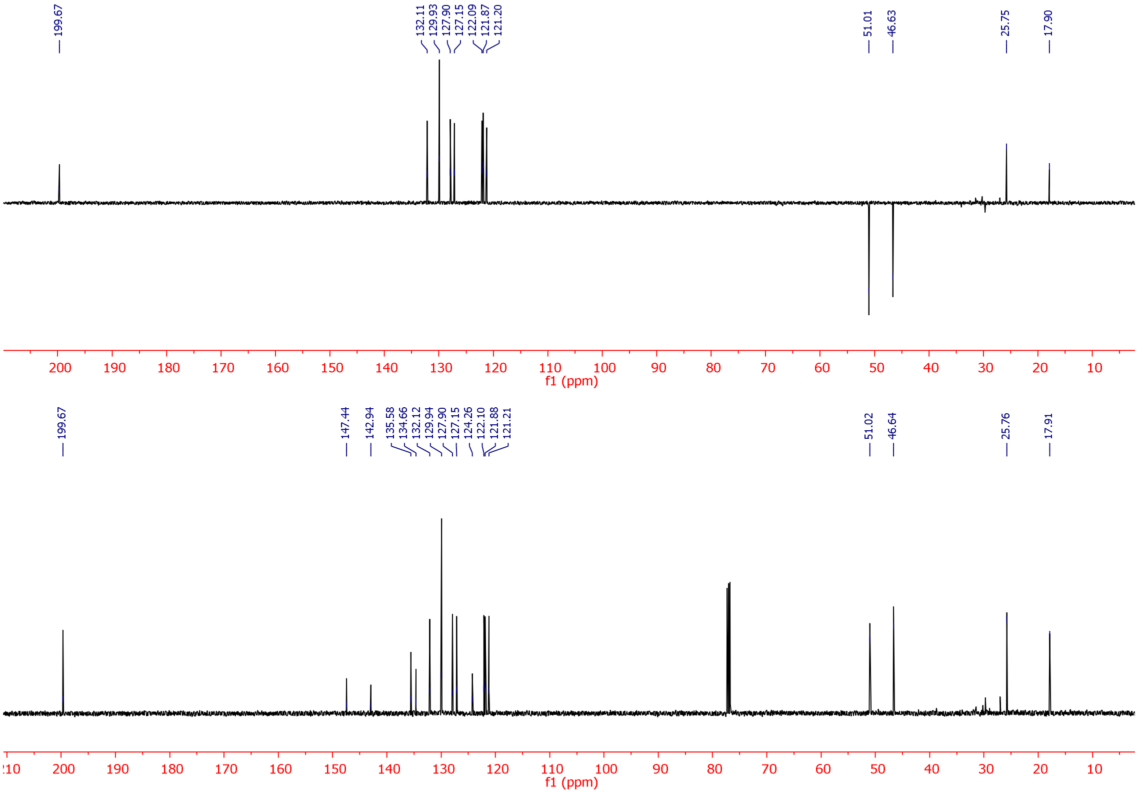

Figure S1c2. ^{13}C NMR spectrum of compound **9b**

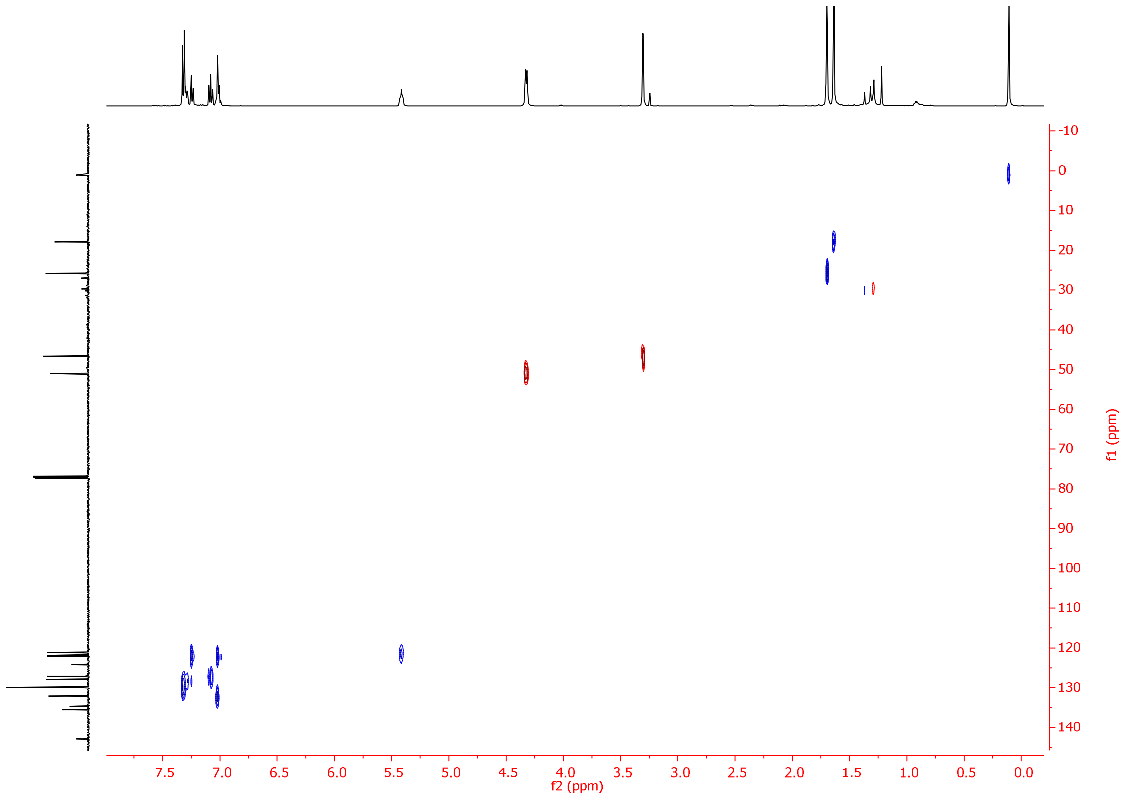

Figure S1d2. HSQC of compound **9b**

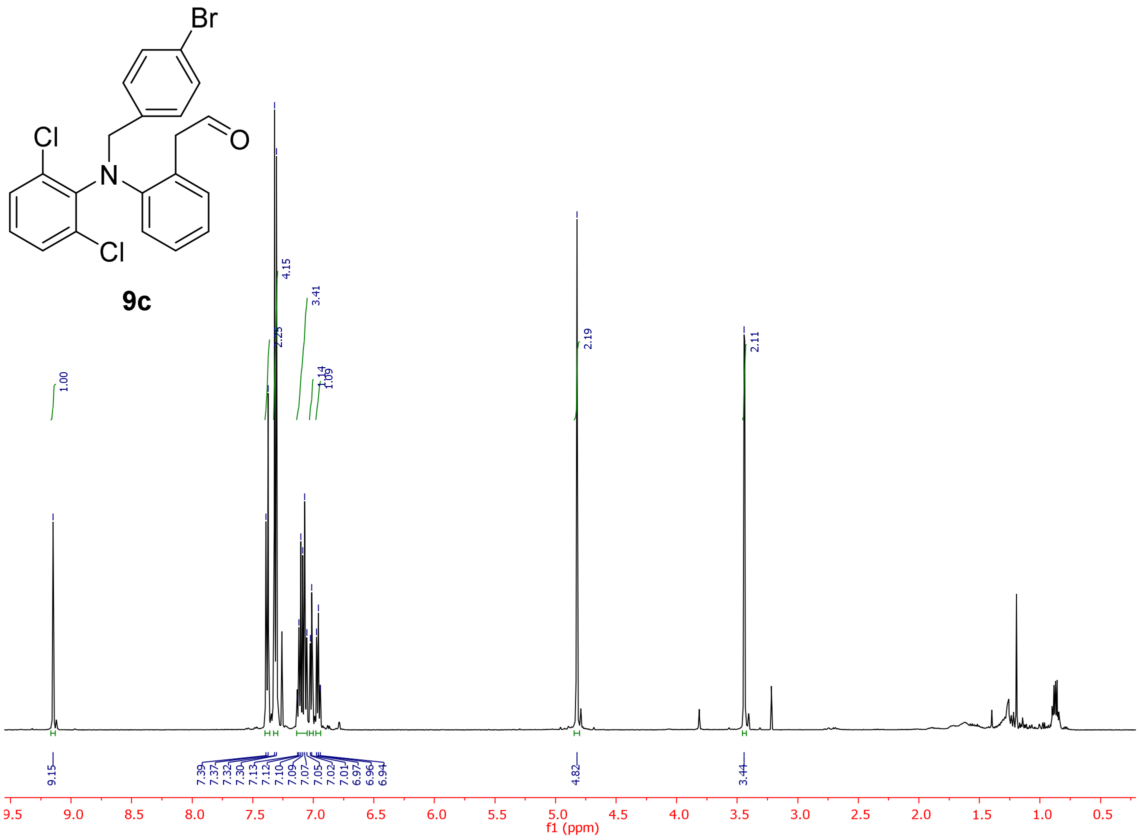

Figure S1e2. ^{1}H NMR spectrum of compound **9c**

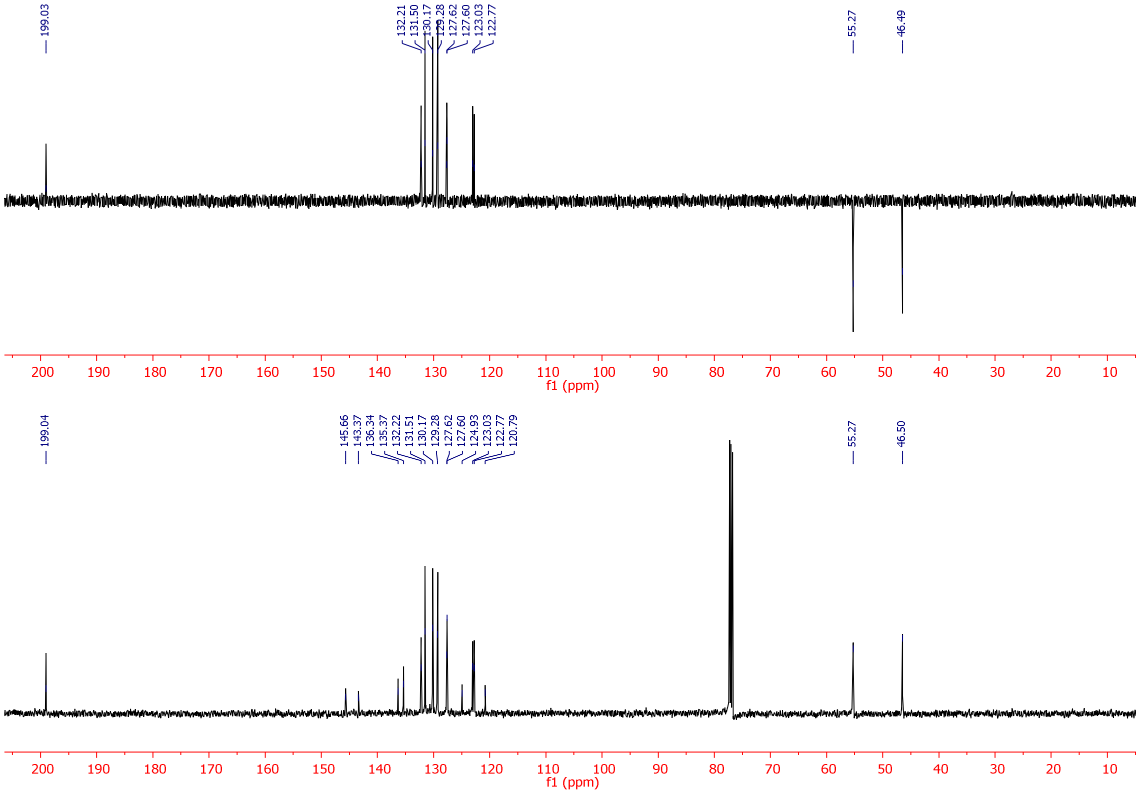

Figure S1f2. ^{13}C NMR spectrum of compound **9c**

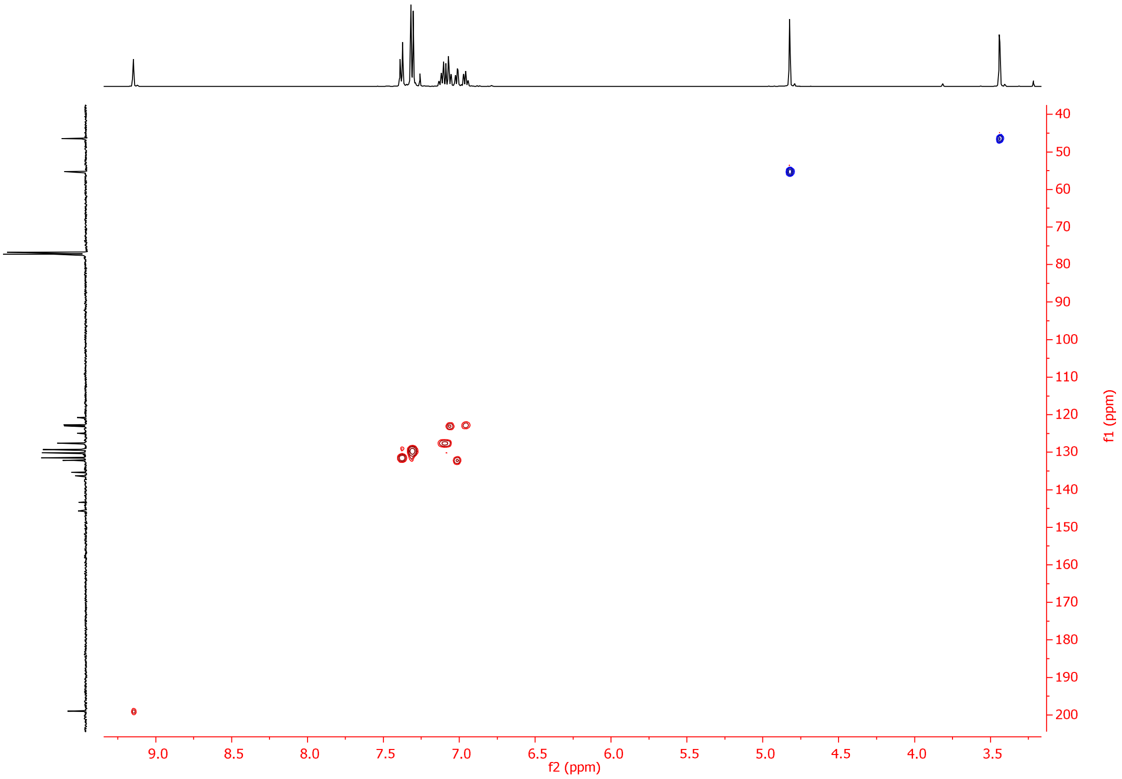

Figure S1g2. HSQC of compound **9c**

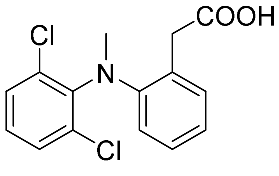

10a

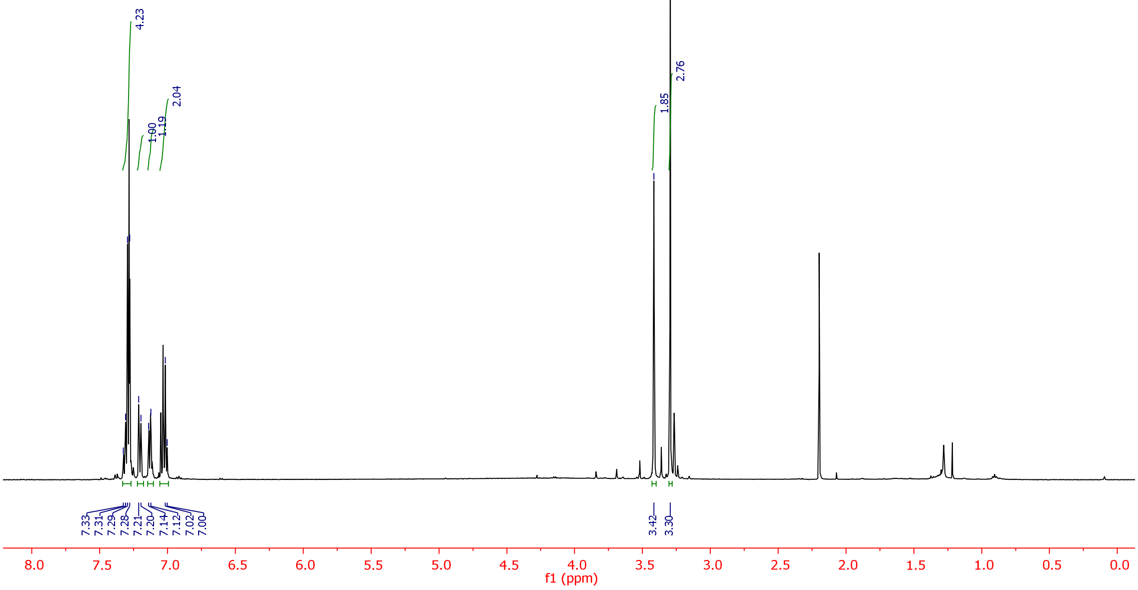

Figure S1h2. ^{1}H NMR spectrum of compound **10a**

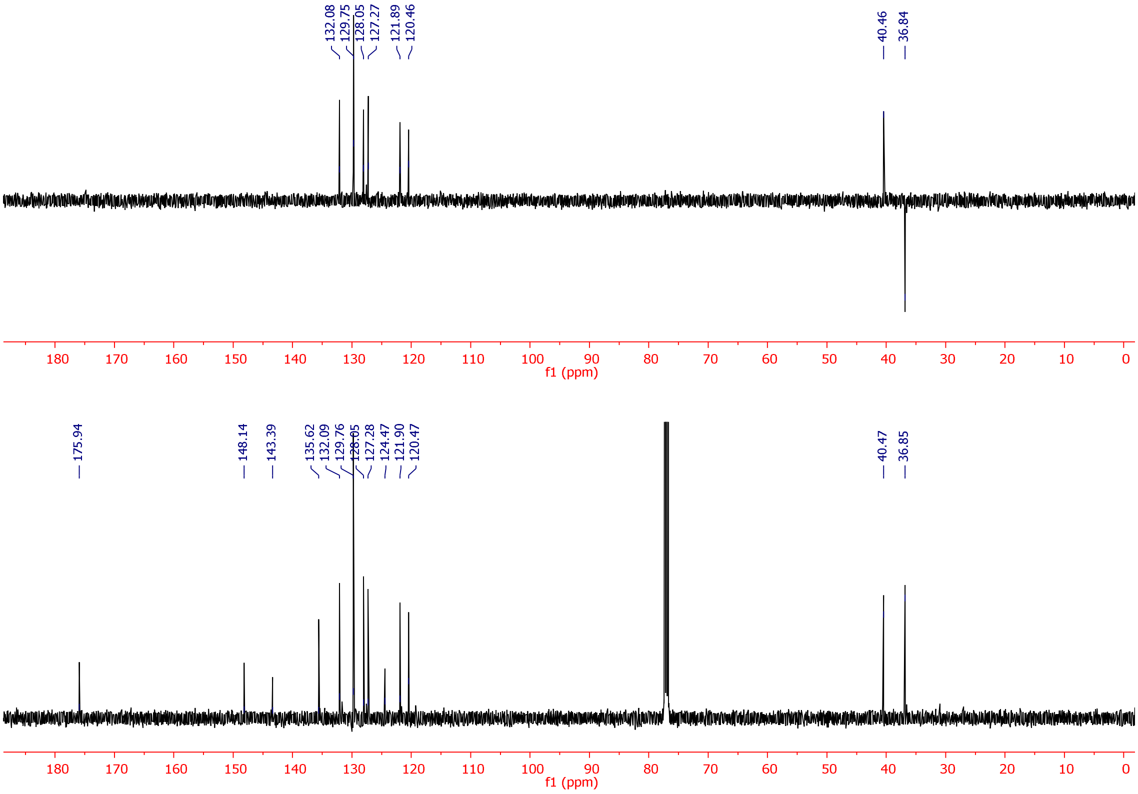

Figure S1i2. ^{13}C NMR spectrum of compound **10a**

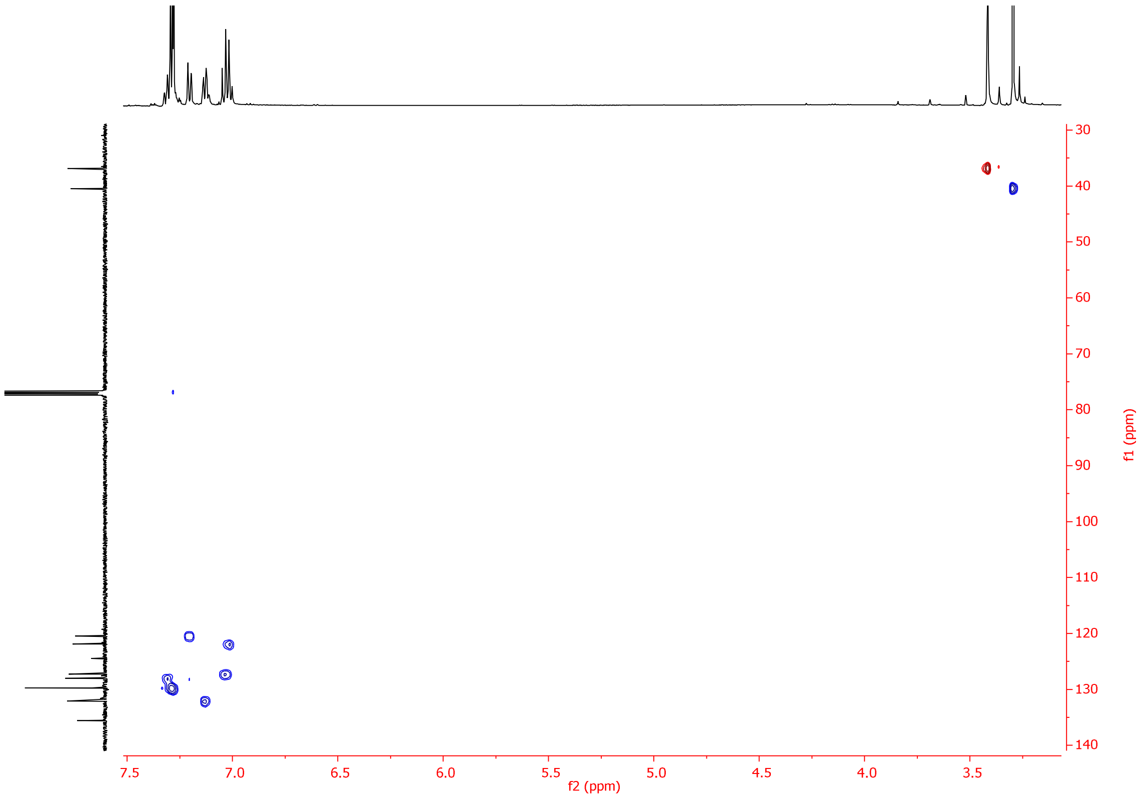

Figure S1j2. HSQC of compound **10a**

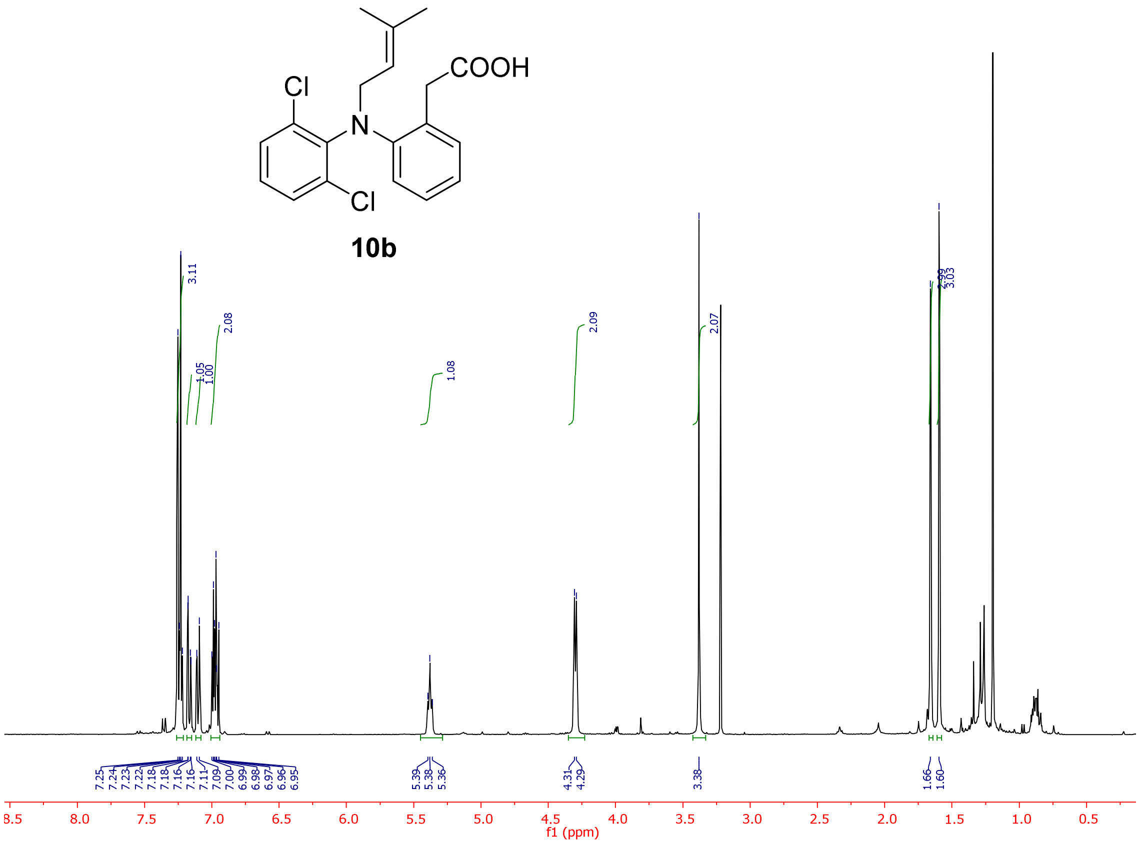

Figure S1k2. ^{1}H NMR spectrum of compound **10b**

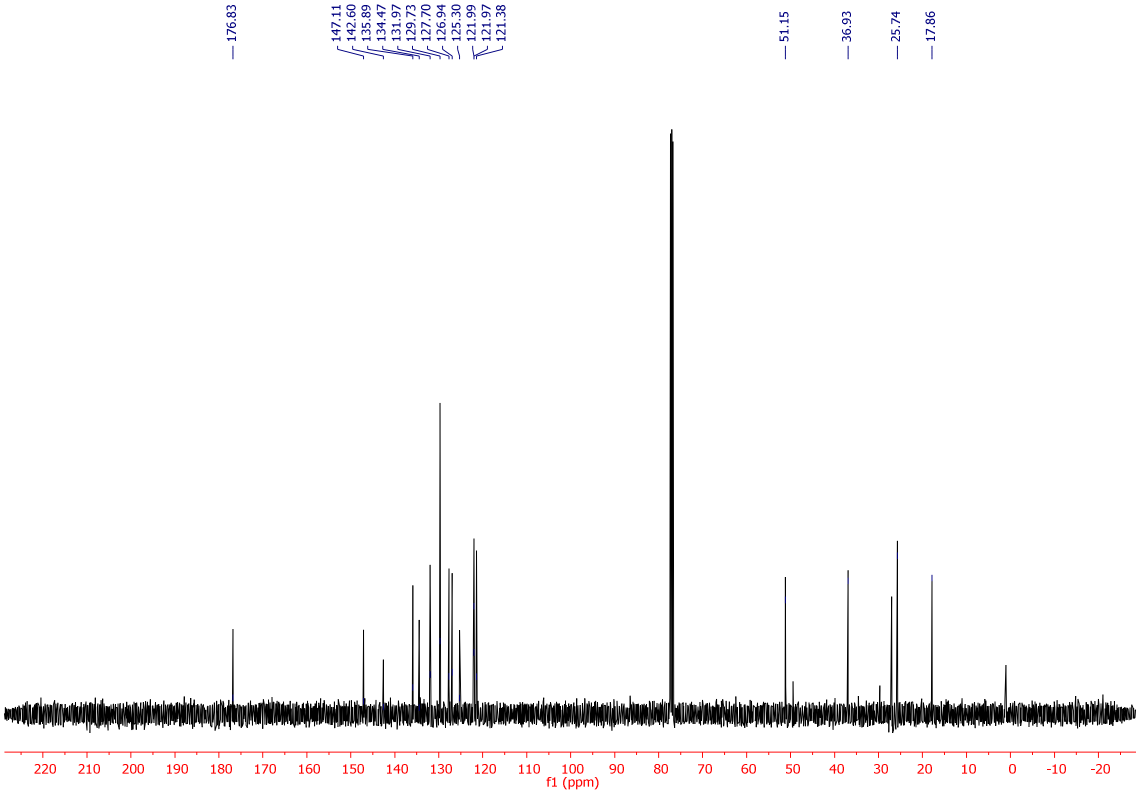

Figure S1l2. ^{13}C NMR spectrum of compound **10b**

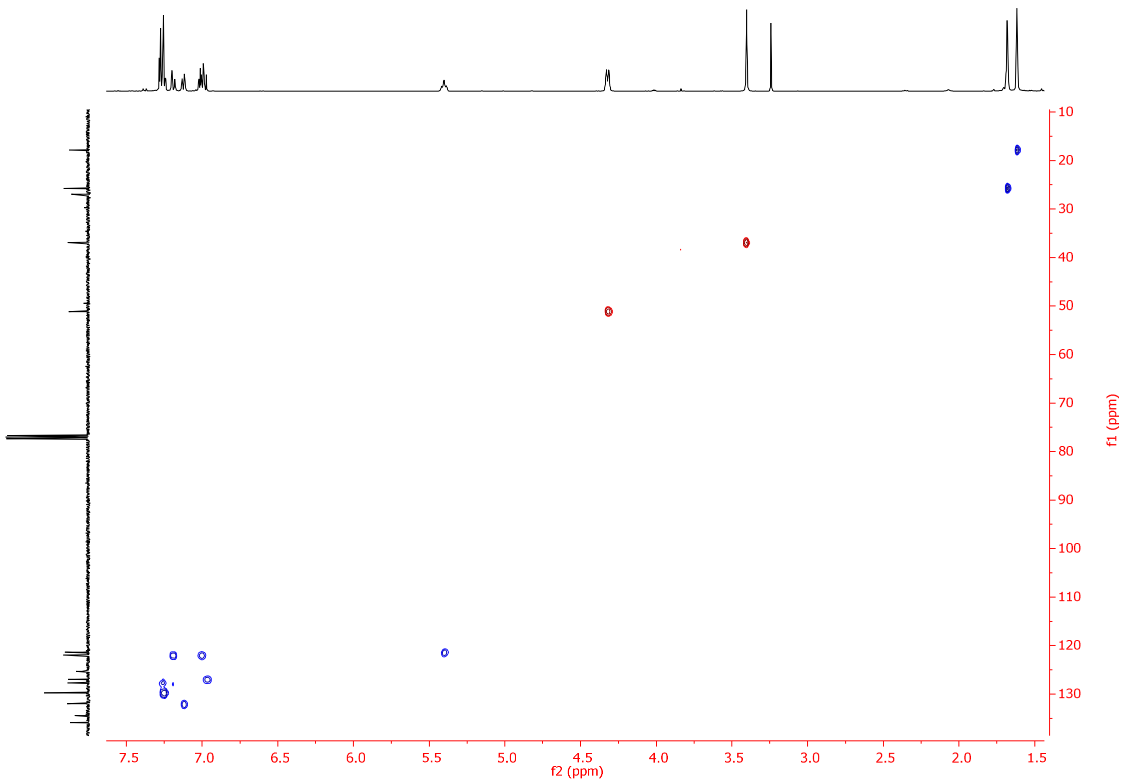

Figure S1j2. HSQC of compound **10b**

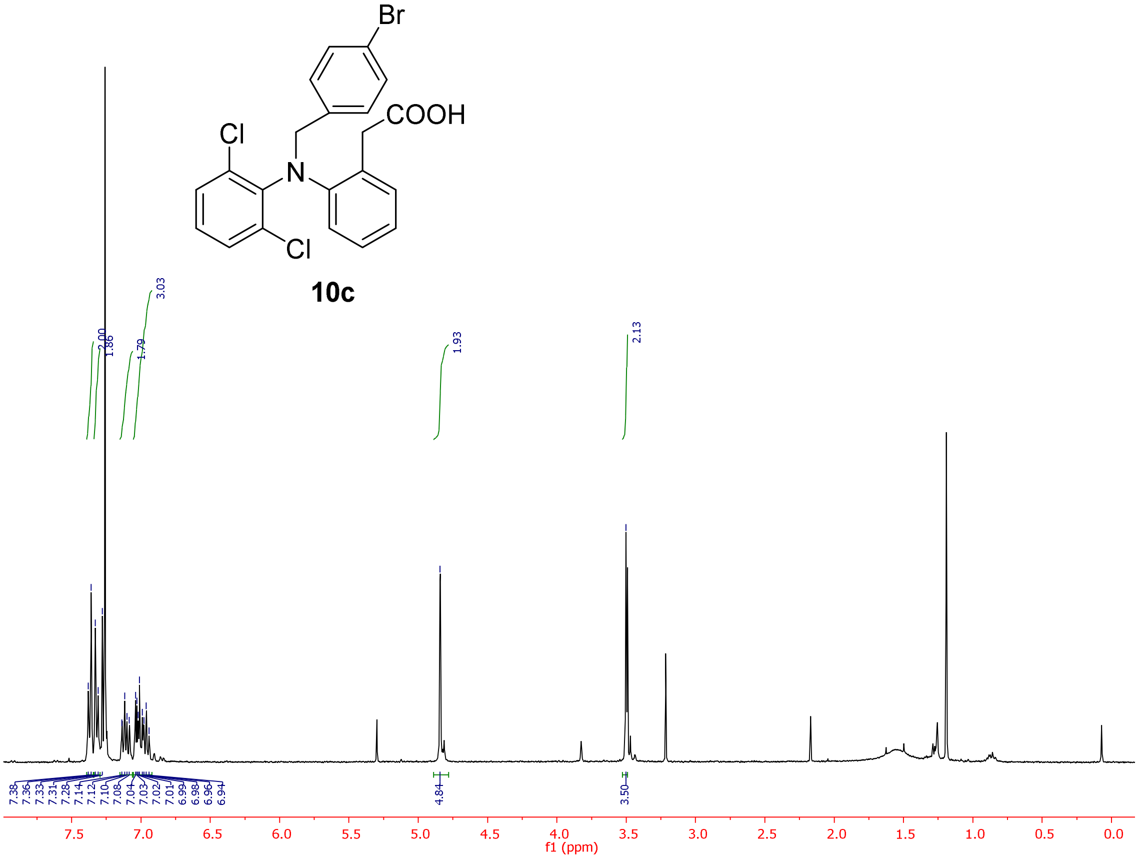

Figure S1l2. ^{1}H NMR spectrum of compound **10c**

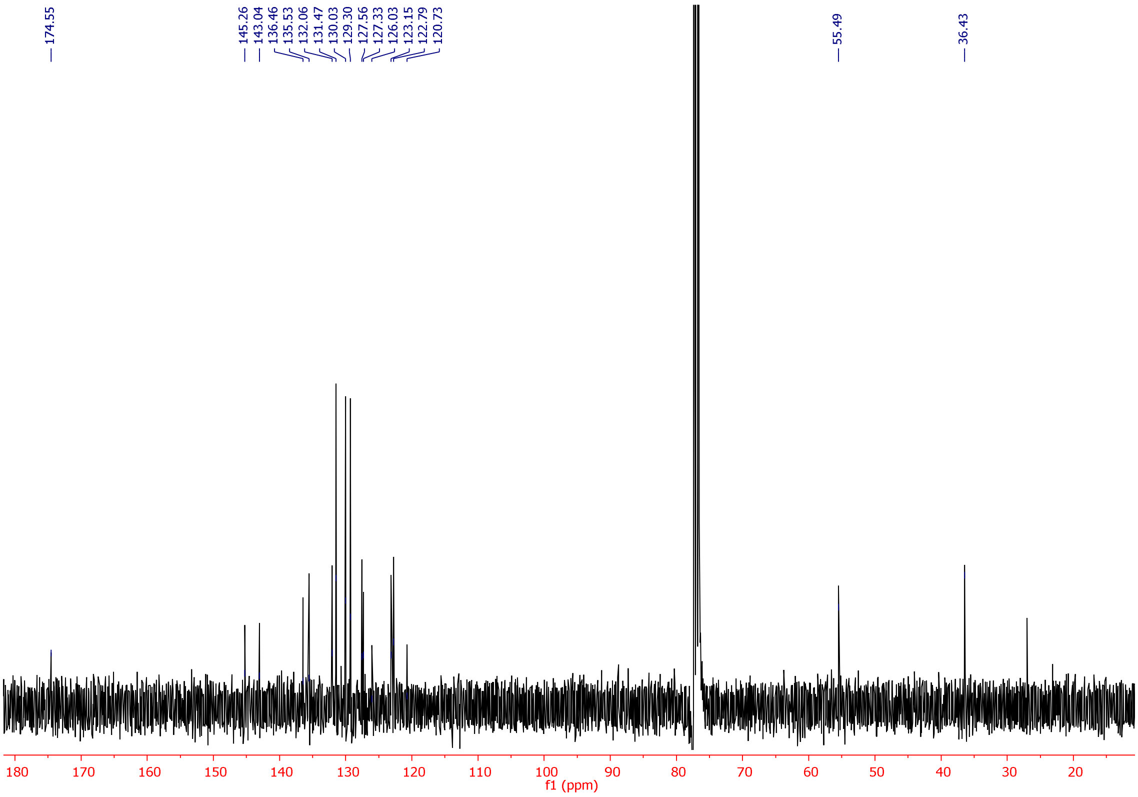

Figure S1m2. ^{13}C NMR spectrum of compound **10c**

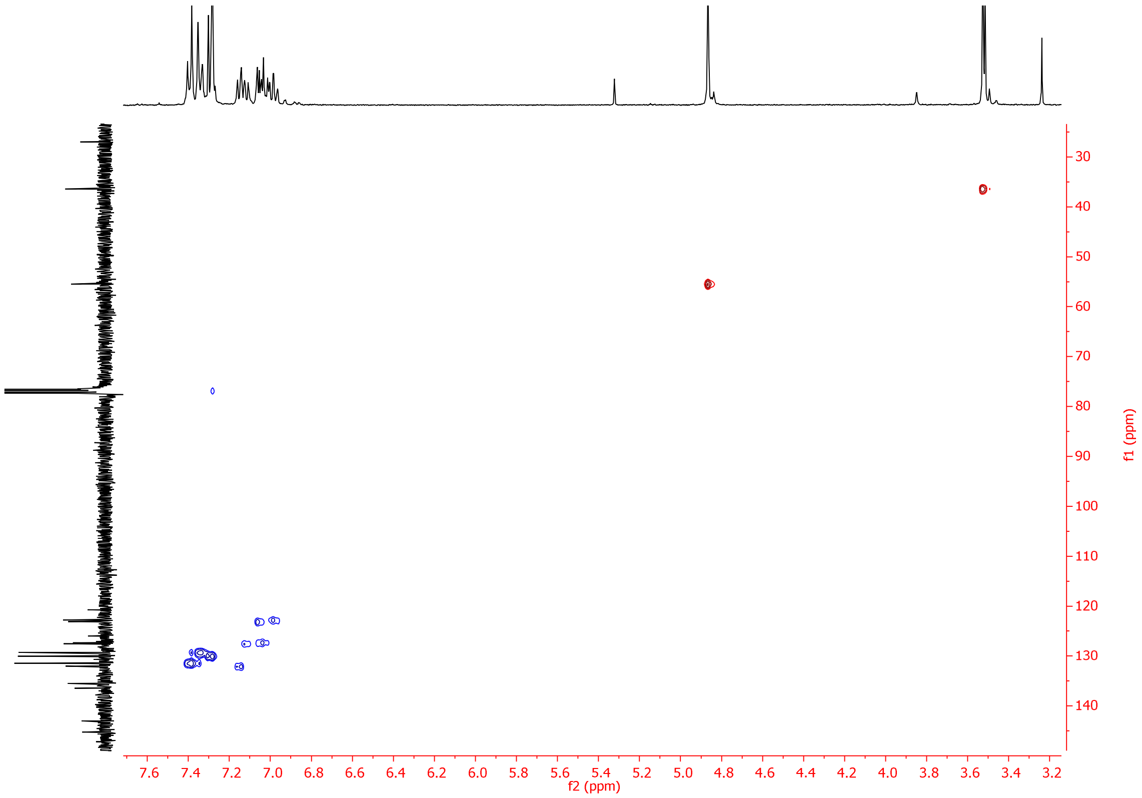

Figure S1n2. HSQC of compound **10c**
